# A Comparative Molecular Dynamics Study of Food-Derived Compounds as PD-L1 Inhibitors: Insights Across Six Flavonoid Subgroups

**DOI:** 10.3390/molecules30040907

**Published:** 2025-02-15

**Authors:** Dejun Jiang, Hyuk-Ku Kwon, Oh Wook Kwon, Youngjin Choi

**Affiliations:** 1 Department of Environmental Engineering, Hoseo University, Asan 31499, Republic of Korea; jdejun@hotmail.com (D.J.); hkkwon@hoseo.edu (H.-K.K.); 2 Pet-Loss Center, Hoseo University, Asan 31499, Republic of Korea; hades770@hanmail.net; 3 Department of Food Science & Technology, Hoseo University, Asan 31499, Republic of Korea

**Keywords:** PD-L1, flavonoid, molecular docking, molecular dynamic simulation, cancer, natural compounds

## Abstract

In this study, we investigated the inhibitory potential of 60 flavonoids from six distinct subgroups on the programmed cell death ligand 1 (PD-L1) dimer through molecular docking and dynamics simulations. Using AutoDock Vina for docking, the binding poses and affinities were evaluated, revealing an average binding affinity of −8.5 kcal/mol for the flavonoids. Among them, ginkgetin exhibited the highest binding free energy of −46.73 kcal/mol, indicating a strong interaction with PD-L1, while diosmin followed closely, with −44.96 kcal/mol. Molecular dynamics simulations were used to further elucidate the dynamic interactions and stability of the flavonoid–PD-L1 complexes, with the analyses showing minimal root mean square deviation (RMSD) and favorable root mean square fluctuation (RMSF) profiles for several compounds, particularly formononetin, idaein, and neohesperidin. Additionally, contact number and hydrogen bond analyses were performed, which highlighted ginkgetin and diosmin as key flavonoids with significant binding interactions, evidenced by their stable conformations and robust molecular interactions throughout the simulations. Ultimately, a cell-based assay confirmed their ability to inhibit the proliferation of cancer cells. These results, validated through cell-based assays, indicate that the strategy of identifying natural compounds with anticancer activity using computational modeling is highly effective.

## 1. Introduction

Programmed death ligand 1 (PD-L1) is a membrane protein that is prominently expressed on tumor cell surfaces, where it binds to the programmed death 1 (PD-1) receptor on T cells [1,2,3]. This interaction inactivates T cells, reducing their ability to recognize and target tumor cells [4]. The inhibition of PD-1/PD-L1 binding can restore T cell function, enhancing the antitumor activity and leading to tumor cell destruction [5,6]. As a result, PD-L1 has become an important target in cancer immunotherapy, prompting research into inhibitors that boost the immune response to cancer cells [7,8].

PD-1/PD-L1-blocking antibodies have shown therapeutic effectiveness in treating cancers such as non-small-cell lung cancer, melanoma, bladder carcinoma, and gastric cancer [9,10]. Currently, six monoclonal antibodies—Nivolumab, Pembrolizumab, Avelumab, Atezolizumab, Cemiplimab, and Durvalumab—are FDA-approved as PD-1/PD-L1 inhibitors [11,12]. However, the widespread clinical use of these antibodies is limited by their high production costs, low tissue penetration, and varied patient responses [13,14], highlighting the need for alternative strategies to inhibit the PD-1/PD-L1 interaction [15,16].

Small-molecule inhibitors represent a promising alternative, offering benefits such as improved stability, enhanced tumor penetration, and reduced side effects compared to antibody therapies [17,18]. Triaryl scaffold compounds, such as BMS-202 and BMS-1166, which were developed by Bristol Myers Squibb, have shown strong potential to inhibit the PD-1/PD-L1 interaction by inducing PD-L1 dimerization [19]. While synthetic small-molecule inhibitors offer notable advantages, an alternative and similarly effective strategy involves identifying functional compounds from plant-derived natural products, leveraging their structural diversity and potential bioactivity as PD-1/PD-L1 pathway modulators. These natural products are gaining attention as potential checkpoint inhibitors due to their complex structures, multitargeting capabilities, and reduced toxicity, making them promising candidates for long-term immunotherapy [20,21]. Although most small-molecule PD-L1 inhibitors, including plant-derived ones, remain in the early developmental stages, further research in this area holds promise in facilitating new, effective cancer therapies [22].

In silico computational methods have become an essential strategy in drug discovery, including the identification of PD-L1 inhibitors. Methods such as molecular docking have been employed to derive the conformations of protein–ligand complexes and generate pharmacophore hypotheses using the 3D crystal structure of human PD-L1 co-crystallized with small-molecule inhibitors, including BMS-202 [23]. Researchers have implemented drug repurposing strategies that combine pharmacophore modeling, molecular docking, and molecular dynamics simulations to identify FDA-approved drugs as potential PD-L1 inhibitors, such as mirabegron and indacaterol, which have shown promising binding affinities and stability with PD-L1 [24]. These methods have advanced the discovery of novel PD-L1 inhibitors by enabling the evaluation of the binding energies of potential inhibitors through computational strategies [25].

To investigate the potential of natural compounds as PD-L1 inhibitors, a comprehensive computational study was conducted to compare the inhibitory effects of six flavonoid subgroups on PD-L1. In the study’s initial stage, 60 flavonoids—10 from each of the flavonol, flavan-3-ol, flavone, isoflavone, anthocyanidin, and flavanone subgroups—were selected for analysis [26,27]. Molecular docking studies provided insights into the flavonoids’ initial binding affinities and binding poses with PD-L1. From each subgroup, the top five compounds identified during docking were further analyzed through molecular dynamics simulations to evaluate their long-term interactions with PD-L1 in a dynamic setting. Post-molecular dynamics (MD) analyses of the simulation trajectories were then performed to gain deeper insights into their binding mechanisms. This multi-tier computational approach revealed the inhibitory potential of flavonoids from different subgroups, highlighting the various approaches of these subgroups in targeting the PD-L1 dimer structure.

## 2. Results and Discussion

### 2.1. Molecular Docking Studies

Molecular docking studies were conducted to assess the initial binding affinities and poses of 60 flavonoids across six subgroups with the PD-L1 dimer using AutoDock Vina, focusing specifically on the hydrophobic pocket. Each subgroup included ten flavonoids, and two known PD-L1 inhibitors from Bristol Myers Squibb (BMS-202 and BMS-1166) were included as references for comparison. As summarized in Table 1, the flavonoids demonstrated an overall average binding affinity of −8.5 kcal/mol. Among the subgroups, flavones and isoflavones displayed the highest average affinities at −9.0 kcal/mol, suggesting greater static binding potential, whereas flavan-3-ols had the lowest affinity, averaging −7.3 kcal/mol. The intermediate-affinity subgroups, namely flavonols, anthocyanidins, and flavanones, showed average docking affinities of −8.9 kcal/mol, −7.9 kcal/mol, and −8.7 kcal/mol, respectively.

As shown in Table 1, the individual docking scores revealed significant binding affinities for ginkgetin and galangin among the flavonols, with values of −10.5 kcal/mol and −10.4 kcal/mol, respectively. These results are close to those of the reference compounds, BMS-202 and BMS-1166, which showed binding affinities of −10.9 kcal/mol and −10.6 kcal/mol, respectively. This suggests that flavones, isoflavones, and specific flavonols, particularly ginkgetin and galangin, may be particularly effective for PD-L1 binding under static conditions. Notably, these top candidates nearly matched the static binding efficacy of the BMS reference compounds in the initial docking assessments.

**Figure 1:**
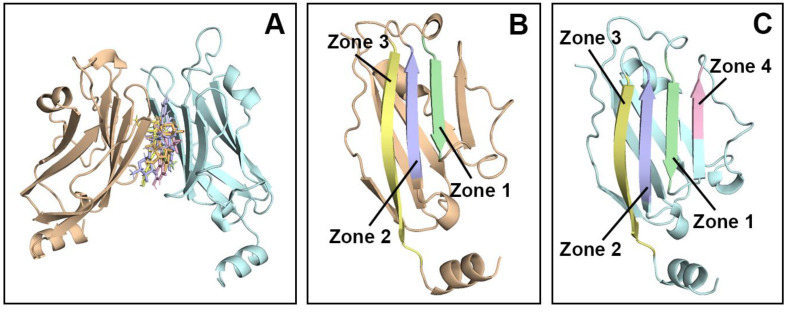
(**A**) The three-dimensional structure of the PD-L1 dimer associated with leading flavonoids from every subgroup; (**B**) chain A of the PD-L1 dimer, highlighting its hydrophobic regions (Zones 1, 2, and 3); (**C**) chain B of the PD-L1 dimer, displaying its hydrophobic areas (Zones 1, 2, 3, and 4).

Figure 1 illustrates the hydrophobic pocket within the PD-L1 dimer, formed by regions from both chain A and chain B. Chain A comprises three hydrophobic zones: Zone 1, spanning residues 36–42; Zone 2, including residues 94–100; and Zone 3, covering residues 104–115. Additionally, chain B contributes a hydrophobic region, Zone 4, comprising residues 48–51. Beta-sheets, specifically Zones 1–4, are key areas that function as binding sites for PD-L1 when exposed to solvents and enclosed by T cells and the CC′ loops of PD-1 proteins [28]. Inducing the dimerization of PD-L1 not only hides these beta-sheets in a concealed position from the solvent but also pre-binds these areas on both sides of PD-L1 using small-molecule flavonoids, which will eliminate the possibility of future binding with PD-1 on T cells due to the initial blockage [29]. These hydrophobic areas play a key role in subsequent post-molecular dynamics analyses. 

### 2.2. Molecular Dynamics Simulations

#### 2.2.1. Binding Free Energy Analysis (MM-PBSA)

**Figure 2:**
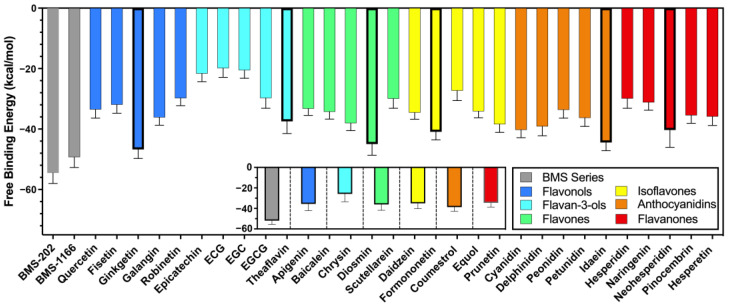
The binding free energy (MM-PBSA) results of the molecular dynamics simulations for subgroups of flavonoids and the BMS series, as well as the overall average binding energy for each subset.

Molecular docking studies are used to assess static interactions, while molecular dynamics simulations provide insights into the dynamic behavior of PD-L1–flavonoid interactions over time, allowing for an examination of the long-term stability of flavonoids with the PD-L1 dimer. MD simulations were conducted on the top five flavonoids that exhibited the highest docking affinities in each subgroup, as detailed in Table 1. Utilizing the Molecular Mechanics Poisson–Boltzmann Surface Area (MM-PBSA) method with the Generalized Born model, the calculation of total binding free energy for these compounds yielded an average binding free energy of −34.47 kcal/mol, as illustrated in Figure 2. This finding suggests that the binding efficiency of these flavonoids is favorable for naturally derived compounds. The calculation of the binding free energy during MD studies utilizes an algorithm to determine the energy difference between a vacuum and solvation. This contrasts the simplified scoring functions adopted in docking studies [30] and ultimately leads to a significant difference between the MM-PBSA results and docking affinities. The correlation between the MM-PBSA and affinities is shown in Figure S1.

Among the subgroups, anthocyanidins exhibited the highest average binding energy at −38.76 kcal/mol, significantly enhancing their performance compared to lower-ranked compounds in the docking studies, indicating improved stability in long-term binding. In contrast, flavan-3-ols recorded the lowest average binding energy at −25.86 kcal/mol, consistent with their poor docking affinity ratings and suggesting less efficient binding to the PD-L1 dimer. Flavonols, flavones, isoflavones, and flavanones demonstrated moderate performance, with average binding free energies of −35.62 kcal/mol, −36.11 kcal/mol, −35.05 kcal/mol, and −35.40 kcal/mol, respectively.

Among the individual flavonoids, ginkgetin exhibited the highest binding free energy at −46.73 kcal/mol, underscoring its strong affinity for PD-L1, as reflected in its docking score. In contrast, epicatechin gallate (ECG) demonstrated the lowest binding energy at −19.88 kcal/mol, with other catechins showing similarly low values. This low average binding energy suggests that catechins may not be optimal candidates for PD-L1 inhibitors, despite some studies exploring their potential in this role [31,32,33].

The reference compounds from Bristol Myers Squibb, such as BMS-202, displayed notable binding energies, reaching a peak of −54.53 kcal/mol. This indicates that ginkgetin could act as an effective inhibitor for PD-L1, comparable to BMS-202. Other leading individual flavonoids from each subgroup, besides ginkgetin, included theaflavin, diosmin, formononetin, idaein, and neohesperidin, which yielded MM-PBSA results of −37.45 kcal/mol, −44.96 kcal/mol, −40.90 kcal/mol, −44.47 kcal/mol, and −40.3 kcal/mol, respectively. After obtaining these findings, extensive post-MD analyses were conducted on these high-performing flavonoids from each subgroup, focusing on deriving insights into their interactions.

#### 2.2.2. Molecular Interaction Analysis

A molecular interaction analysis was conducted to examine the PD-L1 dimer and its interactions with the flavonoids under static conditions. Flavonoids possess a core structure consisting of two aromatic rings and one heterocyclic ring, with minor variations across subgroups. Their distinctions primarily arise from the differences in their extended structures. 

**Figure 3:**
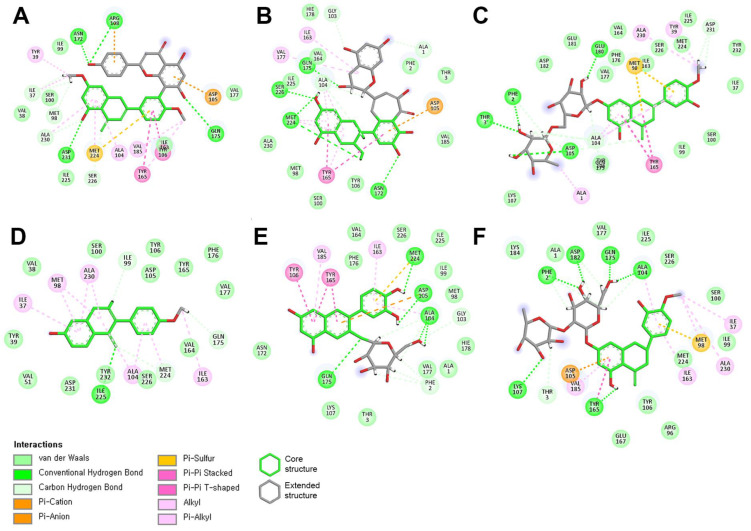
Insights into molecular interactions of top-tier flavonoids: (**A**) ginkgetin, (**B**) theaflavin, (**C**) diosmin, (**D**) formononetin, (**E**) idaein, and (**F**) neohesperidin.

Figure 3 illustrates how flavonoids interact with neighboring residues through a variety of interaction types, including hydrogen bonds (both conventional and carbon), π–ion interactions (π–cation and π–anion), π–π interactions (stacked and T-shaped), alkyl interactions, π–sulfur interactions, and van der Waals forces. The three-ring core structure enables various flavonoids to form multiple π connections within the hydrophobic pocket of the PD-L1 dimer. Notably, some extended structures, such as ginkgetin—a biflavonoid with a dual-core structure—demonstrated enhanced interactions through π connections, while other flavonoids exhibited minimal π interactions within their extended structures.

**Figure 4:**
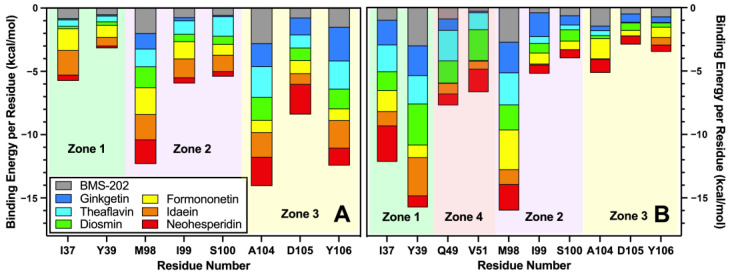
The binding free energies of key residues in chains A (**A**) and B (**B**) of the PD-L1–flavonoid complexes, along with their associated hydrophobic regions.

As illustrated in Figure 4, the per-residue free energy analysis identified key residues that significantly impacted the binding free energy of the PD-L1 dimer–flavonoid complexes in chains A and B. In chain A, critical residues included Ile37 and Tyr39 from Zone 1, as well as Met98, Ile99, and Ser100 from Zone 2, along with Ala104, Asp105, and Tyr106 from Zone 3. For chain B, the notable residues comprised Ile37 and Tyr39 from Zone 1 and Gln49 and Val51 from Zone 4, in addition to Met98, Ile99, and Ser100 from Zone 2 and Ala104, Asp105, and Tyr106 from Zone 3. This indicates that chain B contains a greater number of active residues involved in flavonoid interactions, likely due to the presence of an additional Zone 4. BMS-202 is known to bind to the Ig-like V-type domain of both chains in the PD-L1 dimer, termed Zone 1–4 in this study [34]. In the MM-PBSA approach, BMS-202 contributes binding energy by binding to key residues in this area. As shown in Figure 4, six top-tier flavonoids from each subgroup share significant interactions with PD-L1 in chains A and B, indicating that these flavonoids can implement a similar binding mechanism to the BMS series, despite the differences in their chemical structures, while also offering the advantage of reducing side effects through lower toxicity [35]. According to Figure 3 and Figures S2–S4, the core structure is the dominant binding part of each flavonoid, while the extended structure hinders the drug from entering the hydrophobic pockets. Ginkgetin and diosmin, with their unique cores and extended structures, can travel deeper into the binding site and engage in similar binding interactions to those of BMS-202. This is considered a key reason that these flavonoids exhibit higher binding free energies, approaching those of the BMS series.

In chain A, Met98, Ala104, and Tyr106 emerged as the main contributors among these residues, with all flavonoids exhibiting a notable energy contribution. As detailed in Table 2, Met98 from Zone 2 contributes to the binding energy through one conventional hydrogen bond and two π–alkyl interactions. Ala104, located in Zone 3, enhances this contribution with two conventional hydrogen bonds and three carbon hydrogen bonds, underscoring its significant role. Lastly, Tyr106 from Zone 3 primarily contributes through two π–π interactions and three van der Waals forces.

In chain B, Ile37, Tyr39, and Met98 were identified as the key residues with substantial binding energy contributions. As illustrated in Table 2, Ile37 from Zone 1 contributed through five π–alkyl interactions, while Tyr39 from Zone 1 added one conventional hydrogen bond and five π–π interactions. Additionally, Met98 from Zone 2 contributed to the binding free energy through two conventional hydrogen bonds and one carbon hydrogen bond, highlighting its significant impact in chain B.

#### 2.2.3. RMSD

**Figure 5:**
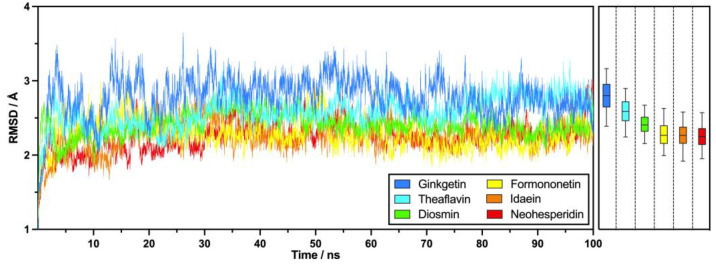
RMSDs from 100 ns MD simulations of key flavonoids across six subgroups, featuring a 5–95% box-and-whisker plot.

Root mean square deviation (RMSD) calculations were performed for the PD-L1 dimer–flavonoid complexes to evaluate the temporal evolution of the structural stability of the PD-L1 dimer during interactions with flavonoids. Initially, the RMSD values increased from 1 Å in the first 5 ns of the simulation, subsequently stabilizing between 2 and 3 Å, as illustrated in Figure 5. The PD-L1–flavonoid complexes exhibited reasonable equilibrium states throughout the molecular dynamics simulations. 

Figure 5 demonstrates that formononetin, idaein, and neohesperidin achieved lower average RMSD values of 2.28 Å, 2.26 Å, and 2.25 Å, respectively, indicating a more stable conformational state among these flavonoids. In contrast, ginkgetin recorded the highest average RMSD at 2.78 Å, despite its strong performance in the MM-PBSA analysis, suggesting comparatively higher backbone fluctuations. The mid-tier flavonoids, theaflavin and diosmin, exhibited RMSD values of 2.58 Å and 2.41 Å, respectively. Throughout the simulation, formononetin, idaein, and neohesperidin consistently maintained low RMSD values with minor fluctuations, while ginkgetin demonstrated higher values with a broader range of variation.

Ginkgetin and diosmin, identified as potential candidates with high binding energy in this study, showed larger deviations than the other flavonoids, highlighting the interactional differences in these compounds within the hydrophobic pocket of the PD-L1 dimer structure. Ginkgetin and diosmin exhibited leading contact numbers during the 100 ns production phase; this will be detailed later. This indicates that, although these compounds have greater conformational flexibility than the others, this flexibility occurs mainly within the hydrophobic pocket. This suggests that there are various favorable binding modes, which, along with their high binding free energies, position these two flavonoids as promising inhibitor candidates [36]. To understand the impacts of the simulation duration on the RMSD, an extended 50 ns production phase was performed, as shown in Figure S5. 

#### 2.2.4. RMSF

**Figure 6:**
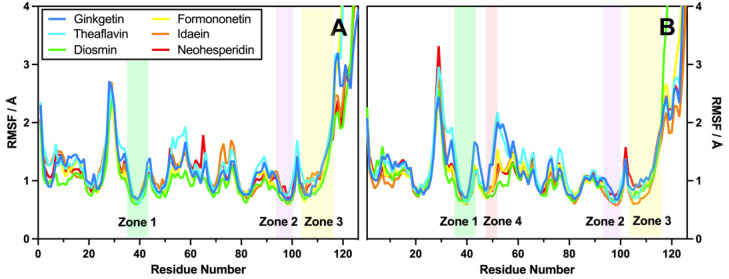
RMSFs of the 100 ns MD simulations for chains A (**A**) and B (**B**) in various PD-L1–flavonoid complex systems, with hydrophobic zones highlighted.

Root mean square fluctuation (RMSF) analyses were conducted on the PD-L1–flavonoid complexes to assess the flexibility of the residues in chains A and B of the PD-L1 dimer. As shown in Figure 6, the six flavonoids exhibited closely aligned RMSF values, primarily ranging between 0.8 and 1.5 Å, suggesting that the PD-L1 dimer–flavonoid systems were relatively stable across all flavonoids. In the hydrophobic regions of both chains, the RMSF values decreased to as little as 0.62 Å, indicating minimal residue flexibility in these critical binding areas. This finding also suggests that there is a strong interaction between the two PD-L1 chains, with the flavonoids serving as connectors. 

Notable differences were observed in the region spanning residues 52–66 in both chains, located shortly after Zone 4, which consists of three turns linked by two coils. Ginkgetin and theaflavin demonstrated less stability compared to the other flavonoids, which may negatively impact their overall conformational stability. The peak RMSF values were identified around residues 29, 45, 52, 77, and 102 in both chains, with corresponding RMSF values of 2.71 Å, 1.39 Å, 1.45 Å, 1.55 Å, and 1.70 Å, respectively, indicating that these residues were relatively sensitive to fluctuations during the simulation. The final coil residue, Pro116A, connects the terminal alpha-helix residues 117-126, primarily contributing to the rising RMSF values observed in the last few residues. RMSF values exceeding 4 Å were excluded from the analysis due to their limited relevance. 

#### 2.2.5. *R*_g_/SASA

**Figure 7:**
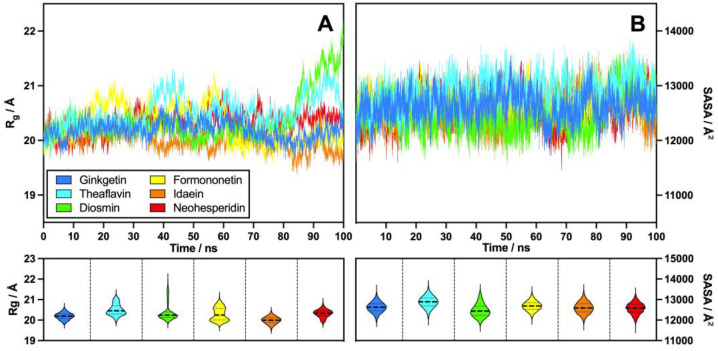
The radius of gyration (**A**) and the solvent-accessible surface area (**B**) of flavonoids from six subgroups, with violin plots highlighting their moderate variability.

Calculations of the radius of gyration (*R*_g_) and the solvent-accessible surface area (SASA) of the PD-L1 dimer–flavonoid complex systems were performed to assess their structural compactness and solvent exposure. As illustrated in Figure 7, the average *R*_g_ values ranged from 19.5 Å to 20.5 Å, indicating that the systems maintained a stable conformational state and demonstrated consistent folding. The median *R*_g_ values for the flavonoids included were 20.19 Å for ginkgetin, 20.45 Å for theaflavin, 20.24 Å for diosmin, 20.24 Å for formononetin, 20.00 Å for idaein, and 20.34 Å for neohesperidin, highlighting the strong alignment in the data. During the simulations, theaflavin and diosmin exhibited signs of unfolding at 40 ns and 90 ns, with corresponding *R*_g_ values of 21.2 Å and 21.9 Å, respectively. Additionally, diosmin demonstrated an increase in *R*_g_ after 85 ns, suggesting a decreased degree of folding at these times. The *R*_g_ values for the various flavonoids clustered around specific values; however, theaflavin and formononetin exhibited a notably wider distribution compared to the other flavonoid groups. Although the system had undergone pre-equilibration at a constant volume and pressure and completed a 100 ns production run, some compounds, especially diosmin and theaflavin, still showed significant conformational changes in the final period, as shown in Figure 7. This indicates that these compounds may require a longer equilibration period, along with additional model parameter settings, to achieve a relatively stabilized conformation [37].

The SASA analysis results, presented in Figure 7, indicate that the flavonoids exhibited greater alignment than the radius of gyration, reflecting the more analogous solvent-exposed areas among these compounds. The median SASA values for the flavonoids were as follows: 12,622 Å^2^ for ginkgetin, 12,888 Å^2^ for theaflavin, 12,437 Å^2^ for diosmin, 12,686 Å^2^ for formononetin, 12,592 Å^2^ for idaein, and 12,583 Å^2^ for neohesperidin. This resulted in an overall average of 12,639.5 Å^2^. Notably, theaflavin displayed a slightly larger solvent-accessible surface area, suggesting that it may have undergone more significant conformational changes compared to the other flavonoids. 

#### 2.2.6. Contact Number Analysis

**Figure 8:**
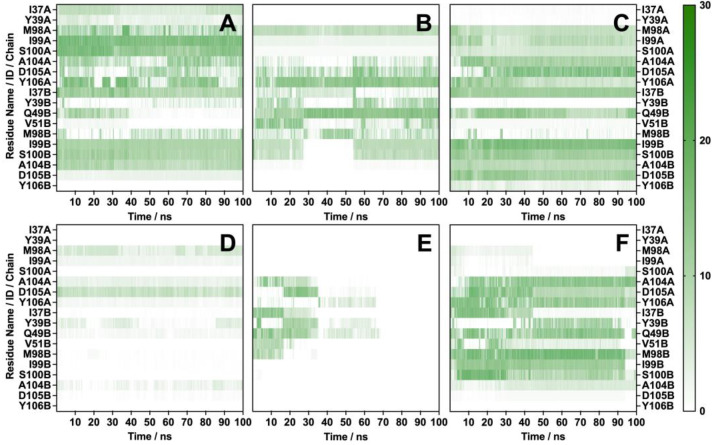
Number of contacts between PD-L1 dimer and flavonoids at crucial residues found in hydrophobic zones: (**A**) ginkgetin, (**B**) theaflavin, (**C**) diosmin, (**D**) formononetin, (**E**) idaein, and (**F**) neohesperidin.

The contact numbers were calculated to assess the frequency in various PD-L1 dimer–flavonoid systems at a distance of 4.5 Å. This analysis revealed notable differences in the interaction counts. As illustrated in Figure 8, ginkgetin had the highest average contact count, reaching 335.7 interactions per frame with the PD-L1 dimer, primarily due to its interactions with I99A and S100A, indicating robust and active engagement. The contact numbers varied significantly, particularly for formononetin, which interacted with residues from Zone 3 of chain A and Zones 1 and 4 of chain B during the initial 40 ns of the simulation. However, it lost most of its contacts by 100 ns, leading to an average of 29.4 interactions per frame, suggesting that formononetin may be less effective for long-term PD-L1 inhibition. Additionally, idaein displayed relatively few interactions, averaging 64.7 contacts per frame, primarily involving key residues M98A and D105A. Flavonoids with moderate numbers of contacts included theaflavin, diosmin, and neohesperidin, which had average contact counts of 262.2, 287.5, and 209.7 per frame, respectively.

#### 2.2.7. Hydrogen Bond Analysis

**Figure 9:**
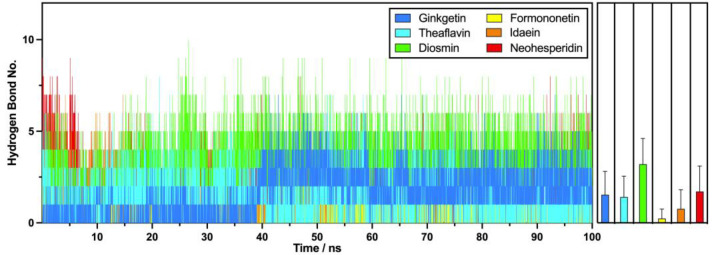
Numbers of hydrogen bonds with the PD-L1 dimer in six leading flavonoids from each subgroup, alongside the average number of hydrogen bonds.

Hydrogen bond analyses were performed on various flavonoid–PD-L1 dimer complexes to investigate the H-bond interactions between the ligands and proteins. As illustrated in Figure 9, diosmin exhibited the highest number of hydrogen bonds with the PD-L1 dimer, averaging 3.2 H-bonds during the 100 ns MD simulation, while formononetin had the lowest average at 0.2 H-bonds. Intermediate numbers of hydrogen bond contacts were observed for ginkgetin, theaflavin, idaein, and neohesperidin, yielding average contact numbers of 1.5, 1.4, 0.8, and 1.7, respectively. In the time-based analysis, ginkgetin and diosmin initially formed relatively few H-bonds in the first 40 ns, but these numbers began to increase thereafter. Conversely, formononetin and idaein started with a few bonds but ultimately lost them all during the 100 ns simulation. Theaflavin and neohesperidin maintained stable H-bonds throughout the simulation, indicating a consistent interaction with the PD-L1 dimer via hydrogen bonds.

**Figure 10:**
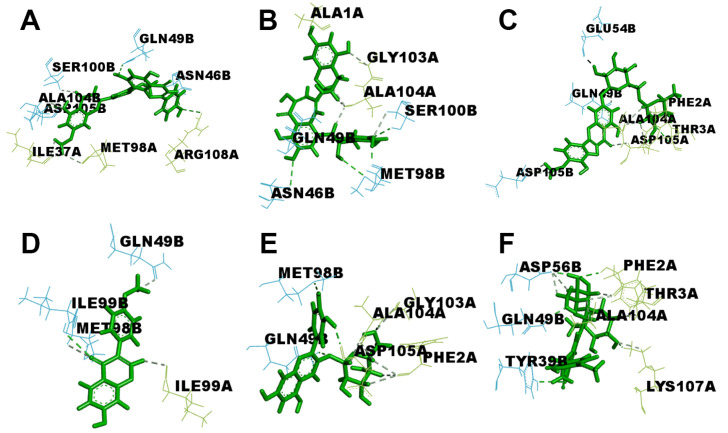
PD-L1 potential residues forming hydrogen bonds with flavonoids: (**A**) ginkgetin, (**B**) theaflavin, (**C**) diosmin, (**D**) formononetin, (**E**) idaein, and (**F**) neohesperidin.

As illustrated in Figure 10, each flavonoid possesses seven to eight potential residues at PD-L1 that form hydrogen bonds, while formononetin has only four. Most of these residues originate from the key hydrophobic zones of the PD-L1 dimer. Details of the bonding interactions are displayed in Figure 10.

#### 2.2.8. Principal Component Analysis

**Figure 11:**
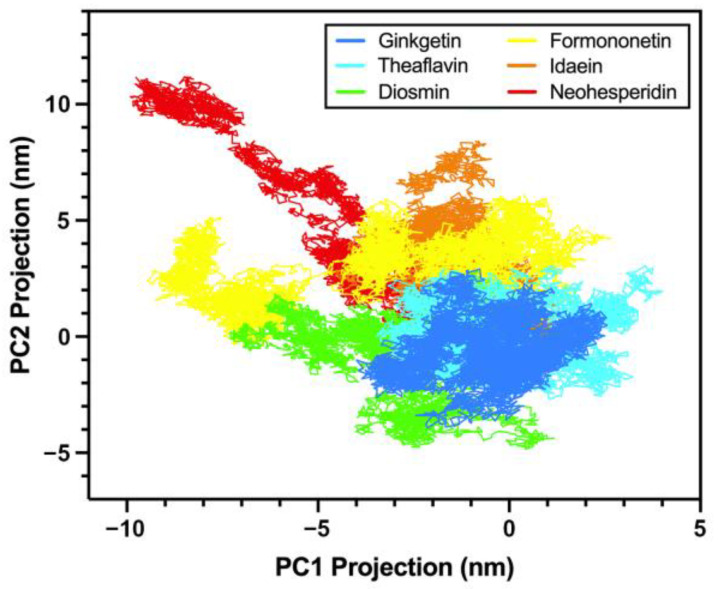
Principal component analysis of flavonoids’ conformational dynamics when interacting with the PD-L1 dimer.

A principal component analysis (PCA) was performed on the different flavonoid systems to examine the conformational dynamics by exploring the free energy landscape. As illustrated in Figure 11, most flavonoids exhibited similarly focused basins, primarily located within −5 to 5 nm in both the PC1 and PC2 projections. However, formononetin and neohesperidin deviated significantly from this region, reaching −10 nm in PC1 and 10 nm in PC2, indicating less compact conformational clusters.

**Figure 12:**
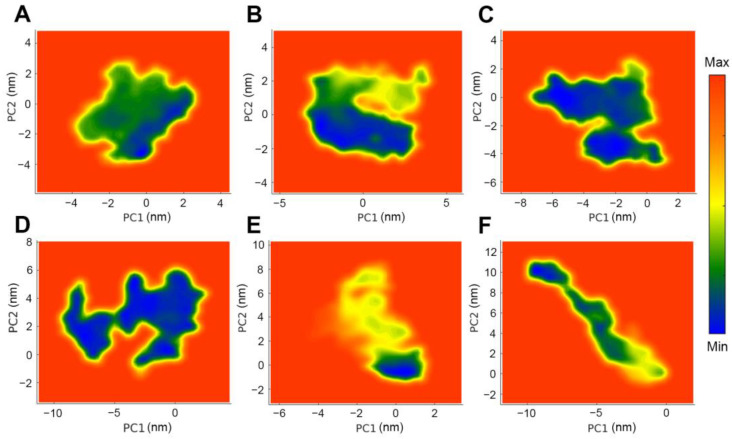
Free energy landscapes of six top-tier flavonoids with the PD-L1 dimer: (**A**) ginkgetin, (**B**) theaflavin, (**C**) diosmin, (**D**) formononetin, (**E**) idaein, and (**F**) neohesperidin.

In Figure 12, deeper colors (blue) indicate that the flavonoid systems achieve a lower energy state, which corresponds with the minimum energy of PD-L1 and its ligand. Throughout the simulation, ginkgetin and theaflavin maintained generally stable conformations. In contrast, diosmin and formononetin exhibited strong yet varied dynamics in their conformations, showing substantial changes in the projections across several nanometers, indicative of relatively unstable conformational behavior. Regarding idaein and neohesperidin, there were not only various conformational shifts but also notable energy changes, typically characterized by significant increases in energy, which aligned with the contact number analysis.

#### 2.2.9. ADMET Predictions

ADMET predictions were utilized to evaluate the clinical potential and drug-likeness of six flavonoids. As indicated in Table 3, these flavonoids displayed variability in gastrointestinal absorption, with formononetin and ginkgetin leading the way, exhibiting high absorption rates of 96.12% and 95.38%, respectively, while diosmin and neohesperidin were anticipated to exhibit relatively low absorption in the gastrointestinal tract. All six flavonoids were predicted to demonstrate negative results for blood–brain barrier permeability and CYP3A4 inhibition, whereas formononetin was predicted to exhibit positive results for both parameters. No compound was expected to show positive results for excretion and toxicity, underscoring the low toxicity characteristic of food-derived flavonoids. Lipinski’s rule of five (RO5) and pan-assay interference compounds (PAINS) were used to obtain predictions regarding the drug-likeness and potential of the compounds considered as candidates for PD-L1 inhibitors in clinical trials. In these assessments, ginkgetin and formononetin were predicted to show no alerts in both RO5 and PAINS. Although ADMET does not guarantee the feasibility and safety of potential candidates as PD-L1 inhibitors, the superior results, particularly for ginkgetin, emphasize the favorable drug-likeness among the six flavonoids [38].

#### 2.2.10. MTT Assay

**Figure 13:**
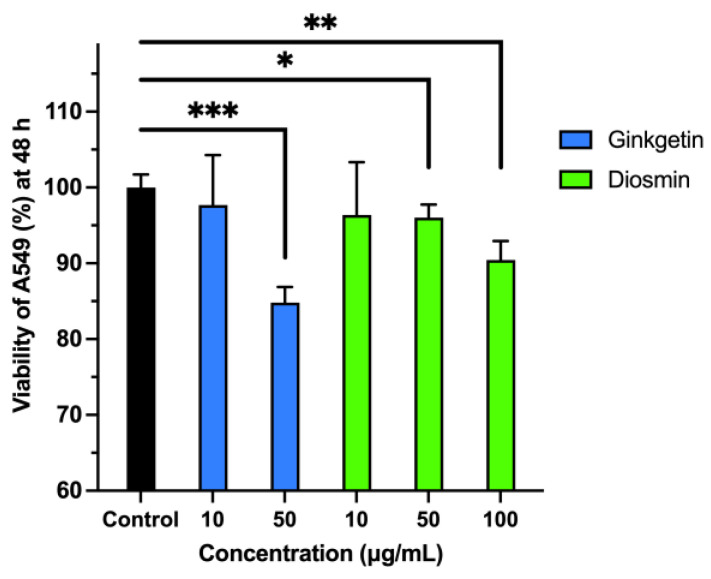
Effects of ginkgetin and diosmin on viability in A549 cells when administered at 10 and 50 μg/mL (ginkgetin) or 10, 50, and 100 μg/mL (diosmin) for 48 h; * *p* < 0.05, ** *p* < 0.01, and *** *p* < 0.001 vs. control.

The cytotoxic effects of ginkgetin and diosmin on cancer cells were assessed through the MTT assay. Figure 13 shows the impacts of these compounds on the viability of A549 lung cancer cells after a 48-h treatment period. Treatment with ginkgetin at 10 µg/mL and 50 µg/mL resulted in a cell viability of 97.69% and 84.16%, respectively. In comparison, diosmin at concentrations of 10 µg/mL, 50 µg/mL, and 100 µg/mL reduced the cell viability to 97.03%, 94.06%, and 90.10%, respectively, indicating that ginkgetin was more effective. These results indicate that ginkgetin and diosmin, both natural compounds, exhibit notable pro-apoptotic properties and may serve as effective inhibitors of PD-L1, thereby influencing cancer cell survival.

In addition to inhibiting PD-L1, flavonoids are widely known for their various secondary effects during cancer therapy due to their anti-inflammatory and antioxidant properties. A study on ginkgetin has shown that it has the ability to inhibit the expression of cytokines, including TNF-α and IL-6, through its anti-inflammatory properties, which helps to reduce side effects during cancer therapy [39]. In addition, its antioxidant properties allow it to mitigate oxidative stress by inhibiting reactive oxygen species (ROS), which helps to relieve oxidative side effects as well [40]. Studies have also indicated that diosmin has the ability to inhibit ABCB1, while formononetin inhibits MRP1 and MRP2. These domains can enhance chemosensitivity, leading to an increase in sensitivity to chemotherapy by maintaining high drug concentrations [41,42]. 

Using the cytotoxic method of the MTT assay to evaluate the clinical potential of ginkgetin and diosmin presents limitations in terms of providing insights into the interactions between flavonoids and PD-L1, as well as in PD-1/PD-L1 interactions. In the future, the complete mapping of the ability of these flavonoids to induce PD-L1 dimerization will require experiments using surface plasmon resonance (SPR) and flow cytometry-based binding assays [43]. The further application of cytokine profiling assays could provide insights into the secondary effects exerted by different flavonoids on cancer cells during cancer therapy through their anti-inflammatory action [44].

## 3. Materials and Methods

### 3.1. Protein and Chemical Structure Preparation

The 3D structure of the PD-L1 dimer (PDB ID: 7DY7) was obtained from the RCSB Protein Data Bank for use in molecular docking and molecular dynamics studies [45]. This structure, which was resolved by Cheng Y. et al. through X-ray diffraction at a resolution of 2.42 Å, was selected due to its greater completeness, with fewer missing atoms and alternate locations, despite its relatively lower resolution compared to other available PD-L1 dimer structures [46].

A total of 60 flavonoid compounds from six subgroups—flavonols, flavans, flavones, isoflavones, anthocyanidins, and flavanones—were selected for this study, as detailed in Table 1 and Figures S6 and S7 [47]. The 2D structures of these flavonoids were sourced from PubChem and used for subsequent analysis [48]. For reference, small-molecule compounds from Bristol Myers Squibb, known for their PD-L1-inhibitory capabilities, were included as benchmark structures [49,50]. The reference structures, BMS-202 and BMS-1166, were also acquired from PubChem for comparative purposes.

PD-L1 structure cleansing was performed using PyMOL 3.0.3 to eliminate water and ligand molecules [51]. To obtain the 3D structures of the flavonoids, RDKit and Open Babel were utilized to facilitate format conversion from PDB to PDBQT and 3D structure generation [52,53]. Finally, the Kollman charges and hydrogen atoms were added to the PD-L1 dimer structure using MGLTools 1.5.7 for molecular docking [54].

### 3.2. Molecular Docking

A diverse range of molecular docking programs are currently available for researchers, including AutoDock, AutoDock Vina, DockThor, and GOLD [54,55,56,57]. In this study, we conducted molecular docking studies using AutoDock Vina 1.1.2, which is renowned for its excellent scoring accuracy and balanced computational efficiency. It was used to explore the binding affinity and initial binding sites of various flavonoids on the PD-L1 dimer [55]. The grid box center was positioned based on the original binding site of ligand Compound 17 in the PD-L1 dimer structure (PDB ID: 7DY7). The grid coordinates were set to 144.2 Å, −13.2 Å, and 19.8 Å for the X, Y, and Z axes, respectively, with a cubic search area of 20 Å per side, encompassing the hydrophobic pocket between chains A and B of the PD-L1 dimer. For each flavonoid, the binding pose with the lowest binding affinity was visualized and analyzed using VMD and Discovery Studio [58,59].

Various strategies were applied to validate the accuracy of the molecular docking studies. Binding region validation was achieved through the web-based PrankWeb [60]. The grid box center and box size were validated by redocking the original ligand of 7DY7 to the PD-L1 dimer, as depicted in Figure S8. The discriminative ability of the docking model was assessed by generating a receiver operating characteristic (ROC) curve using 60 flavonoids examined in this study, along with 60 flavonoid-like decoys generated with the web-based DUDE server, as presented in Figure S9 [61]. Ultimately, as shown in Figure S10, the docking affinities of the BMS series were compared to the experimental IC_50_ values obtained from the studies conducted by Guzik, K. et al. in 2017 [50].

### 3.3. Molecular Dynamics Simulations

Molecular dynamics simulations were conducted using AMBER 22 and AMBERTools 23 in a Linux environment, aiming to assess the long-term stability and interactions between the PD-L1 dimer and various flavonoid subgroups [62]. The 3D structures of the flavonoids were refined using the AM1-BCC method in the Antechamber tool to assign partial atomic charges [63]. Each system was solvated within an OPC octahedral water box with a 10 Å buffer and neutralized with Na^+^ ions using the LEaP tool in AMBERTools [64]. The simulations were performed with the AMBER ff19SB force field, selected for its compatibility with the OPC water model, to ensure the accurate representation of protein–water interactions throughout [65].

A 5000-cycle CPU-based minimization procedure was conducted to optimize the initial complex system to a lower energy state. This included 2500 cycles using the steepest descent method, followed by 2500 cycles of conjugate gradient minimization. Following minimization, a 20 ps CPU-based heating phase using the Langevin thermostat method was applied, gradually raising the system’s temperature from 100 K to 310 K under constant-volume (NVT) conditions. A subsequent 20 ps constant-pressure (NPT) equilibration step was then conducted to allow a smooth transition between the thermodynamic conditions. During the NVT and NPT phases, restraints were applied to the PD-L1 dimer at 50 kcal/mol/Å^2^ and 10 kcal/mol/Å^2^, respectively, to ensure system stability. The production phase comprised a 100 ns simulation, accelerated by two GPUs, under constant pressure, for the top 5 flavonoids with the highest binding affinities in each subgroup [66]. The SHAKE algorithm was applied to constrain the hydrogen bond lengths, while the Monte Carlo barostat was used for pressure control [67,68]. Electrostatic interactions were computed using the Particle Mesh Ewald (PME) method. The temperature during the production phase was maintained at 310 K using a Langevin thermostat in order to replicate physiological conditions. To ensure their accuracy and reproducibility, the molecular dynamics simulations were repeated three times for all flavonoids tested.

### 3.4. Binding Free Energy Calculations

The gas-phase binding free energy was determined by calculating the molecular mechanics (MM) energy difference between the bound complex and the unbound receptor and ligand, as described in Equations (1) and (2) [69]. The enthalpic contributions ($E_{MM}^{0}$) included van der Waals forces and electrostatic interactions, as well as bonded terms such as bond stretching, angle bending, and dihedral torsion energies. Entropic contributions ($S_{normal\;mode\;analysis}^{0}$) were examined using normal mode analysis to explain the entropy change in the binding energy.

(1) $$\Delta G_{bind,vacuum}^{0}=G_{complex,vacuum}^{0}-\left(G_{ligand,vacuum}^{0}+G_{receptor,vacuum}^{0}\right)$$

(2) $$G_{vacuum}^{0}=E_{MM}^{0}-T\times S_{normal\;mode\;analysis}^{0}$$

The solvation free energy was computed to justify solvent effects on the binding. The estimation of the electrostatic contribution in both vacuum and solvent environments was performed using the Generalized Born (GB) model, following Equations (3) and (4) [70]. The difference between the vacuum and solvent values represented the electrostatic solvation free energy ($G_{electrostatic}^{0}$). In addition to the electrostatic contribution, the hydrophobic energy (${\Delta G}_{hydrophobic}^{0}$) was estimated based on the solvent-accessible surface area (SASA).

(3) $${\Delta G}_{bind,solv}^{0}={\Delta G}_{complex,solv}^{0}-({\Delta G}_{ligand,solv}^{0}+{\Delta G}_{receptor,solv}^{0})$$

(4) $${\Delta G}_{solv}^{0}=G_{electrostatic,\epsilon =80}^{0}-G_{electrostatic,\epsilon =1}^{0}+{\Delta G}_{hydrophobic}^{0}$$

The total binding free energy (${\Delta G}_{bind,TOTAL}^{0}$) was obtained by combining the gas-phase energy and the solvation free energy correction, representing the free energy of binding in solution, as outlined in Equation (5).

(5) $${\Delta G}_{bind,TOTAL}^{0}={\Delta G}_{bind,vacuum}^{0}+{\Delta G}_{bind,solv}^{0}$$

### 3.5. Principal Component Analysis Methodology

The principal component analysis (PCA) was undertaken using the cpptraj module within AmberTools, aiming to explore the conformational variability of the system and identify the dominant poses of motion [71]. This technique was employed in order represent the collective motions more simply, seeking to reduce the dimensionality of the data by transforming the residue fluctuations into principal components (PC1, PC2) [72]. This analysis was conducted based on the covariance matrix of atomic displacements, which was used to map the free energy landscape. The calculations focused on C-alpha atoms in all residues in both chains and the flavonoid structure so as to reduce the noise from the side chain effect. Principal components capture the conformational changes in a system and contribute to the later identification of dynamic shifts between different conformational states and the exploration of their relationships with the corresponding free energy profiles. Projections of the trajectory onto these principal components reveal the most relevant motions contributing to the system’s structural dynamics and their potential influences on the binding free energy.

### 3.6. ADMET Analysis

The ADMET parameters were predicted using the web-based servers SwissADME and pkCSM [73,74]. Key parameters, including intestinal absorption, BBB permeability, CYP3A4, renal OCT2 substrates, and AMES toxicity, were anticipated with pkCSM to assess the clinical potential of the flavonoids as PD-L1 inhibitors. SwissADME was used to validate the parameters gathered from pkCSM and to generate drug-likeness predictions based on Lipinski’s rule of five, as well as medicinal chemistry features by assessing PAINS alerts.

### 3.7. Cell Culture and MTT Assay

A549 cells were grown in RPMI-1640 medium, enriched with 10% FBS and 1% PS. They were cultured in a humidified environment at 37 °C with 5% CO_2_ [75]. To evaluate the cell viability, the A549 cells were plated in 96-well plates at a density of 1 × 10^4^ cells per well and treated with different concentrations of the test agents (10, 50, and 100 µg/mL) for 48 h. The 3-[4,5-dimethylthiazol-2-yl]-2,5-diphenyl-tetrazolium bromide (MTT) assay was used to quantitatively measure the cell viability by assessing the metabolic activity of the cells [76].

### 3.8. Statistical Analysis

Data from the experiments are displayed as the mean ± standard error of the mean (S.E.M.). Statistical significance among the different treatment groups was assessed using Student’s *t*-test. Differences were regarded as statistically significant for *p*-values below 0.05. This statistical method guarantees a comprehensive evaluation of the efficacy of the treatments being investigated [77].

## 4. Conclusions

In this study, we comprehensively evaluated the inhibitory potential of 60 flavonoids from six subgroups on the PD-L1 dimer, utilizing molecular docking and dynamics simulations. The docking results provided insights into the binding poses and affinities of the initial flavonoids. Furthermore, the molecular dynamics simulations offered valuable information about the dynamic interactions, revealing that ginkgetin, from the flavonol group, exhibited the highest binding free energy of −46.73 kcal/mol among the six key flavonoids, closely followed by diosmin at −44.96 kcal/mol. In-depth post-MD analyses confirmed the stability and molecular interactions of these PD-L1–flavonoid complexes. These MD-based computational approaches hold great promise regarding their application in the development of natural product-derived therapeutics.

## Figures and Tables

**Table 1:** Overview of 60 flavonoids’ characteristics, including their subgroups, sources, CAS numbers, docking affinities, and delivery status in MD simulations.

Subgroup	No.	Compound	Source	CAS No.	Affinity (kcal/mol)	MD Simulation
Flavonols	1	Quercetin	Apples	117-39-5	−8.7	Yes
	2	Kaempferol	Broccoli	520-18-3	−7.6	No
	3	Myricetin	Walnuts	529-44-2	−8.5	No
	4	Fisetin	Strawberries	528-48-3	−9.5	Yes
	5	Rutin	Buckwheat	153-18-4	−8.4	No
	6	Isorhamnetin	Pears	480-19-3	−8.2	No
	7	Morin	Osage orange	480-16-0	−8.0	No
	8	Ginkgetin	Ginkgo biloba	481-46-9	−10.5	Yes
	9	Galangin	Propolis	548-83-4	−10.4	Yes
	10	Robinetin	Acacia	490-31-3	−9.2	Yes
Flavan-3-ols	11	Catechin	Tea	18829-70-4	−7.3	No
	12	Epicatechin	Dark chocolate	490-46-0	−8.5	Yes
	13	ECG	Green tea	1257-08-5	−8.2	Yes
	14	EGC	Green tea	970-74-1	−8.6	Yes
	15	EGCG	Green tea	989-51-5	−8.5	Yes
	16	Theaflavin	Black tea	4670-05-7	−9.2	Yes
	17	Theaflavin-3-gallate	Black tea	28543-07-9	−7.6	No
	18	Procyanidin B1	Apples	20315-25-7	−7.1	No
	19	Procyanidin B2	Apples	29106-49-8	−6.8	No
	20	Procyanidin C1	Cocoa	37064-30-5	−1.5	No
Flavones	21	Apigenin	Chamomile	520-36-5	−9.0	Yes
	22	Luteolin	Celery	491-70-3	−8.8	No
	23	Baicalein	Chinese skullcap	491-67-8	−10.0	Yes
	24	Chrysin	Honey	480-40-0	−9.9	Yes
	25	Tangeretin	Tangerine	481-53-8	−8.1	No
	26	Nobiletin	Citrus fruits	478-01-3	−8.2	No
	27	Wogonin	Scutellaria baicalensis	632-85-9	−8.4	No
	28	Diosmin	Citrus fruits	520-27-4	−9.6	Yes
	29	Acacetin	Chamomile	480-44-4	−8.9	No
	30	Scutellarein	Scutellaria species	529-53-3	−9.3	Yes
Isoflavones	31	Genistein	Soybeans	446-72-0	−9.0	No
	32	Daidzein	Soybeans	486-66-8	−9.6	Yes
	33	Glycitein	Soy products	40957-83-3	−9.0	No
	34	Formononetin	Red clover	485-72-3	−9.1	Yes
	35	Biochanin A	Red clover	491-80-5	−8.9	No
	36	Sissotrin	Red clover	5928-26-7	−7.7	No
	37	Puerarin	Kudzu vine	3681-99-0	−9.0	No
	38	Coumestrol	Clover	479-13-0	−9.6	Yes
	39	Equol	Soy derivatives	531-95-3	−9.2	Yes
	40	Prunetin	Prunes	552-59-0	−9.1	Yes
Anthocyanidins	41	Cyanidin	Berries	13306-05-3	−8.7	Yes
	42	Delphinidin	Berries	13270-61-6	−7.9	Yes
	43	Malvidin	Grapes	10463-84-0	−7.8	No
	44	Pelargonidin	Strawberries	7690-51-9	−7.9	No
	45	Peonidin	Berries	134-01-0	−7.9	Yes
	46	Petunidin	Berries	1429-30-7	−7.9	Yes
	47	Rosinidin	Rhododendrons	4092-64-2	−7.8	No
	48	Capensinidin	Capensia flowers	19077-85-1	−7.6	No
	49	Callistephin	Strawberries	18466-51-8	−7.5	No
	50	Idaein	Lingonberries	27661-36-5	−8.4	Yes
Flavanones	51	Hesperidin	Citrus fruits	520-26-3	−9.5	Yes
	52	Naringenin	Grapefruits	480-41-1	−9.0	Yes
	53	Eriodictyol	Lemons	552-58-9	−8.8	No
	54	Isosakuranetin	Citrus fruits	480-43-3	−7.1	No
	55	Naringin	Grapefruit	10236-47-2	−8.2	No
	56	Neohesperidin	Citrus fruits	13241-33-3	−9.4	Yes
	57	Sakuranetin	Cherries	2957-21-3	−8.3	No
	58	Sterubin	Yerba santa	51857-11-5	−8.5	No
	59	Pinocembrin	Honey	480-39-7	−9.7	Yes
	60	Hesperetin	Citrus fruits	520-33-2	−8.9	Yes
Inhibitors	61	BMS-202	Bristol Myers Squibb	1675203-84-5	−10.9	Yes
	62	BMS-1166	Bristol Myers Squibb	1818314-88-3	−10.6	Yes

**Table 2:** Varied interaction frequencies of six top-tier flavonoids with essential residues while exhibiting the highest binding free energy during molecular dynamics studies.

Residue	H Bond	C-H Bond	π–Ion	π–π	π–Alkyl	π–Sulfur	van der Waals
I37A	-	1	-	-	3	-	1
Y39A	-	-	-	-	2	-	1
M98A	-	1	-	-	2	1	2
I99A	-	1	-	-	-	-	4
S100A	-	-	-	-	-	-	5
A104A	2	3	-	-	6	-	-
D105A	2	1	4	-	-	-	1
Y106A	-	-	-	2	-	-	3
I37B	-	-	-	-	5	-	2
Y39B	1	-	-	5	2	-	1
Q49B	4	2	-	-	-	-	-
V51B	-	-	-	-	1	-	5
M98B	2	1	-	-	2	2	2
I99B	1	-	-	-	-	-	5
S100B	1	1	-	-	-	-	4
A104B	-	1	-	-	4	-	1
D105B	1	1	-	-	-	-	1
Y106B	-	-	-	-	-	-	2

**Table 3:** ADMET predictions with Lipinski’s rule of 5 and PAINS alerts for ginkgetin, theaflavin, diosmin, formononetin, idaein, and neohesperidin.

Compound	Absorption	Distribution	Metabolism	Excretion	Toxicity		Lipinski’s Rule	PAINS Alerts
	GI Absorption (% Absorbed)	BBB Permeability (log BB)	CYP3A4	OCT2 Substrate	AMES	hERG I		
Ginkgetin	95.38	−1.884	No	No	No	No	0 alert	0 alert
Theaflavin	65.08	−1.729	No	No	No	No	3 alert	1 alert
Diosmin	29.32	−1.795	No	No	No	No	3 alert	0 alert
Formononetin	96.12	0.157	Yes	No	No	No	0 alert	0 alert
Idaein	45.39	−1.713	No	No	No	No	2 alert	1 alert
Neohesperidin	20.65	−1.720	No	No	No	No	3 alert	0 alert

## Data Availability

Data are contained within the article.

## Supplementary Materials

The following are available online: https://www.mdpi.com/article/10.3390/molecules30040907/s1. Figure S1: Correlation between docking affinity and MM-PBSA; Figure S2: Comparison of the binding modes between BMS-202 (gray) and flavonoids (A) ginkgetin and (B) theaflavin; Figure S3: Comparison of the binding modes between BMS-202 (gray) and flavonoids (A) diosmin and (B) formononetin; Figure S4: Comparison of the binding modes between BMS-202 (gray) and flavonoids (A) idaein and (B) neohesperidin; Figure S5: RMSD from extended 50 ns MD simulations of key flavonoids across six subgroups, featuring a 5–95% box-and-whiskers plot of an extended period; Figure S6: Two-dimensional structures of flavonols (No. 1–10), flavan-3-ols (No. 11–20), and flavones (No. 21–30) involved in initial molecular docking studies; Figure S7: Two-dimensional structures of isoflavones (No. 31–40), anthocyanidins (No. 41–50), flavanones (No. 51–60), and BMS series (No. 61, 62) involved in initial molecular docking studies; Figure S8: Comparison of the binding modes of the original (gray) and redocked (pink) Compound 17; Figure S9: Receiver operating characteristic (ROC) curve generated for 60 flavonoids with 60 flavonoid-like decoys; Figure S10: Linear relationship between docking affinities and experimental IC_50_ values.

## References

[1] Yamaguchi H., Hsu J.-M., Yang W.-H., Hung M.-C. (2022). Mechanisms Regulating PD-L1 Expression in Cancers and Associated Opportunities for Novel Small-Molecule Therapeutics. Nat. Rev. Clin. Oncol..

[2] Yi M., Zheng X., Niu M., Zhu S., Ge H., Wu K. (2022). Combination Strategies with PD-1/PD-L1 Blockade: Current Advances and Future Directions. Mol. Cancer.

[3] Huang J., Liu D., Wang Y., Liu L., Li J., Yuan J., Jiang Z., Jiang Z., Hsiao W.W., Liu H. (2022). Ginseng Polysaccharides Alter the Gut Microbiota and Kynurenine/Tryptophan Ratio, Potentiating the Antitumour Effect of Antiprogrammed Cell Death 1/Programmed Cell Death Ligand 1 (Anti-PD-1/PD-L1) Immunotherapy. Gut.

[4] Brahmer J.R., Tykodi S.S., Chow L.Q.M., Hwu W.-J., Topalian S.L., Hwu P., Drake C.G., Camacho L.H., Kauh J., Odunsi K. (2012). Safety and Activity of Anti–PD-L1 Antibody in Patients with Advanced Cancer. N. Engl. J. Med..

[5] Choi J.-G., Kim Y.S., Kim J.H., Kim T.I., Li W., Oh T.W., Jeon C.H., Kim S.J., Chung H.-S. (2020). Anticancer Effect of Salvia Plebeia and Its Active Compound by Improving T-Cell Activity via Blockade of PD-1/PD-L1 Interaction in Humanized PD-1 Mouse Model. Front. Immunol..

[6] Lee E.-J., Kim Y.S., Kim J.H., Woo K.W., Park Y.-H., Ha J.-H., Li W., Kim T.I., An B.K., Cho H.W. (2024). Uncovering the Colorectal Cancer Immunotherapeutic Potential: Evening Primrose (*Oenothera biennis*) Root Extract and Its Active Compound Oenothein B Targeting the PD-1/PD-L1 Blockade. Phytomedicine.

[7] Yan T., Yu L., Shangguan D., Li W., Liu N., Chen Y., Fu Y., Tang J., Liao D. (2023). Advances in Pharmacokinetics and Pharmacodynamics of PD-1/PD-L1 Inhibitors. Int. Immunopharmacol..

[8] Peng Y., Zhang Z., Yang G., Dai Z., Cai X., Liu Z., Yun Q., Xu L. (2024). N6-Methyladenosine Reader Protein IGF2BP1 Suppresses CD8 + T Cells-Mediated Tumor Cytotoxicity and Apoptosis in Colon Cancer. Apoptosis.

[9] Wu B., Huang X., Shi X., Jiang M., Liu H., Zhao L. (2024). LAMTOR1 Decreased Exosomal PD-L1 to Enhance Immunotherapy Efficacy in Non-Small Cell Lung Cancer. Mol. Cancer.

[10] Wang R., Yu Q., Wang X., Zhu D., Li G., Li Z., Jiang W., Li W., Dang Y. (2023). Bis(Benzonitrile) Dichloroplatinum (II) Interrupts PD-1/PD-L1 Interaction by Binding to PD-1. Acta Pharmacol. Sin..

[11] Lin X., Kang K., Chen P., Zeng Z., Li G., Xiong W., Yi M., Xiang B. (2024). Regulatory Mechanisms of PD-1/PD-L1 in Cancers. Mol. Cancer.

[12] Krishnamoorthy H.R., Karuppasamy R. (2023). A Multitier Virtual Screening of Antagonists Targeting PD-1/PD-L1 Interface for the Management of Triple-Negative Breast Cancer. Med. Oncol..

[13] Wang L., Zheng J., Tan Z., Zhang Y., Wang H. (2024). A Novel Bispecific Peptide Targeting PD-1 and PD-L1 with Combined Antitumor Activity of T-Cells Derived from the Patients with TSCC. Int. Immunopharmacol..

[14] Hsu J.-M., Li C.-W., Lai Y.-J., Hung M.-C. (2018). Posttranslational Modifications of PD-L1 and Their Applications in Cancer Therapy. Cancer Res..

[15] Phetphoung T., Malla A., Rattanapisit K., Pisuttinusart N., Damrongyot N., Joyjamras K., Chanvorachote P., Phakham T., Wongtangprasert T., Strasser R. (2022). Expression of Plant-Produced Anti-PD-L1 Antibody with Anoikis Sensitizing Activity in Human Lung Cancer Cells via., Suppression on Epithelial-Mesenchymal Transition. PLoS ONE.

[16] Barnwal A., Das S., Bhattacharyya J. (2023). Repurposing Ponatinib as a PD-L1 Inhibitor Revealed by Drug Repurposing Screening and Validation by In Vitro and In Vivo Experiments. ACS Pharmacol. Transl. Sci..

[17] Koblish H.K., Wu L., Wang L.-C.S., Liu P.C.C., Wynn R., Rios-Doria J., Spitz S., Liu H., Volgina A., Zolotarjova N. (2022). Characterization of INCB086550: A Potent and Novel Small-Molecule PD-L1 Inhibitor. Cancer Discov..

[18] Wang S., Wang Y., Yan H. (2023). Progress on Biphenyl Derivatives as PD-1/PD-L1 Inhibitors. Med. Chem. Res..

[19] Lu L., Qi Z., Wang T., Zhang X., Zhang K., Wang K., Cheng Y., Xiao Y., Li Z., Jiang S. (2022). Design, Synthesis, and Evaluation of PD-1/PD-L1 Antagonists Bearing a Benzamide Scaffold. ACS Med. Chem. Lett..

[20] Guo Y., Liang J., Liu B., Jin Y. (2021). Molecular Mechanism of Food-Derived Polyphenols on PD-L1 Dimerization: A Molecular Dynamics Simulation Study. Int. J. Mol. Sci..

[21] Yang Y., Liu Q., Shi X., Zheng Q., Chen L., Sun Y. (2021). Advances in Plant-Derived Natural Products for Antitumor Immunotherapy. Arch. Pharmacal Res..

[22] Liu C., Seeram N.P., Ma H. (2021). Small Molecule Inhibitors against PD-1/PD-L1 Immune Checkpoints and Current Methodologies for Their Development: A Review. Cancer Cell Int..

[23] Chandrasekaran J., Elumalai S., Murugesan V., Kunjiappan S., Pavadai P., Theivendren P. (2023). Computational Design of PD-L1 Small Molecule Inhibitors for Cancer Therapy. Mol. Divers..

[24] Sobral P.S., Luz V.C.C., Almeida J.M.G.C.F., Videira P.A., Pereira F. (2023). Computational Approaches Drive Developments in Immune-Oncology Therapies for PD-1/PD-L1 Immune Checkpoint Inhibitors. Int. J. Mol. Sci..

[25] Wang Y., Guo H., Feng Z., Wang S., Wang Y., He Q., Li G., Lin W., Xie X.-Q., Lin Z. (2019). PD-1-Targeted Discovery of Peptide Inhibitors by Virtual Screening, Molecular Dynamics Simulation, and Surface Plasmon Resonance. Molecules.

[26] Wang S., Xiong F., Liu Y., Feng Z. (2024). Exploring Flavonoid Intake and All-Cause Mortality in Diverse Health Conditions: Insights from NHANES 2007–2010 and 2017–2018. Nutrition.

[27] Speciani M.C., Cintolo M., Marino M., Oren M., Fiori F., Gargari G., Riso P., Ciafardini C., Mascaretti F., Parpinel M. (2022). Flavonoid Intake in Relation to Colorectal Cancer Risk and Blood Bacterial DNA. Nutrients.

[28] Zak K.M., Grudnik P., Magiera K., Dömling A., Dubin G., Holak T.A. (2017). Structural Biology of the Immune Checkpoint Receptor PD-1 and Its Ligands PD-L1/PD-L2. Structure.

[29] Wu X., Wang N., Liang J., Wang B., Jin Y., Liu B., Yang Y. (2023). Is the Triggering of PD-L1 Dimerization a Potential Mechanism for Food-Derived Small Molecules in Cancer Immunotherapy? A Study by Molecular Dynamics. Int. J. Mol. Sci..

[30] King E., Aitchison E., Li H., Luo R. (2021). Recent Developments in Free Energy Calculations for Drug Discovery. Front. Mol. Biosci..

[31] Wu P., Zhang H., Yin Y., Sun M., Mao S., Chen H., Deng Y., Chen S., Li S., Sun B. (2022). Engineered EGCG-Containing Biomimetic Nanoassemblies as Effective Delivery Platform for Enhanced Cancer Therapy. Adv. Sci..

[32] Rawangkan A., Wongsirisin P., Namiki K., Iida K., Kobayashi Y., Shimizu Y., Fujiki H., Suganuma M. (2018). Green Tea Catechin Is an Alternative Immune Checkpoint Inhibitor That Inhibits PD-L1 Expression and Lung Tumor Growth. Molecules.

[33] Ravindran Menon D., Li Y., Yamauchi T., Osborne D.G., Vaddi P.K., Wempe M.F., Zhai Z., Fujita M. (2021). EGCG Inhibits Tumor Growth in Melanoma by Targeting JAK-STAT Signaling and Its Downstream PD-L1/PD-L2-PD1 Axis in Tumors and Enhancing Cytotoxic T-Cell Responses. Pharmaceuticals.

[34] Andrei S.A., Sijbesma E., Hann M., Davis J., O’Mahony G., Perry M.W.D., Karawajczyk A., Eickhoff J., Brunsveld L., Doveston R.G. (2017). Stabilization of Protein-Protein Interactions in Drug Discovery. Expert Opin. Drug Discov..

[35] Anand David A., Arulmoli R., Parasuraman S. (2016). Overviews of Biological Importance of Quercetin: A Bioactive Flavonoid. Pharmacogn. Rev..

[36] Shi D., An X., Bai Q., Bing Z., Zhou S., Liu H., Yao X. (2019). Computational Insight Into the Small Molecule Intervening PD-L1 Dimerization and the Potential Structure-Activity Relationship. Front. Chem..

[37] Gallo M.T., Grant B.J., Teodoro M.L., Melton J., Cieplak P., Phillips G.N., Stec B. (2009). Novel Procedure for Thermal Equilibration in Molecular Dynamics Simulation. Mol. Simul..

[38] Luo L., Zhong A., Wang Q., Zheng T. (2021). Structure-Based Pharmacophore Modeling, Virtual Screening, Molecular Docking, ADMET, and Molecular Dynamics (MD) Simulation of Potential Inhibitors of PD-L1 from the Library of Marine Natural Products. Mar. Drugs.

[39] Zhang J., Yang S., Chen F., Li H., Chen B. (2017). Ginkgetin Aglycone Ameliorates LPS-Induced Acute Kidney Injury by Activating SIRT1 via Inhibiting the NF-κB Signaling Pathway. Cell Biosci..

[40] Wang Y.-Q., Wang M.-Y., Fu X.-R., Yu P., Gao G.-F., Fan Y.-M., Duan X.-L., Zhao B.-L., Chang Y.-Z., Shi Z.-H. (2015). Neuroprotective Effects of Ginkgetin against Neuroinjury in Parkinson’s Disease Model Induced by MPTP via Chelating Iron. Free Radic. Res..

[41] To K.K.W., Cho W.C.S. (2021). Flavonoids Overcome Drug Resistance to Cancer Chemotherapy by Epigenetically Modulating Multiple Mechanisms. Curr. Cancer Drug Targets.

[42] He M., Xia L., Li J. (2021). Potential Mechanisms of Plant-Derived Natural Products in the Treatment of Cervical Cancer. Biomolecules.

[43] Cheng X., Veverka V., Radhakrishnan A., Waters L.C., Muskett F.W., Morgan S.H., Huo J., Yu C., Evans E.J., Leslie A.J. (2013). Structure and Interactions of the Human Programmed Cell Death 1 Receptor. J. Biol. Chem..

[44] Zhao L., Zhang Q., Ma W., Tian F., Shen H., Zhou M. (2017). A Combination of Quercetin and Resveratrol Reduces Obesity in High-Fat Diet-Fed Rats by Modulation of Gut Microbiota. Food Funct..

[45] Berman H.M. (2000). The Protein Data Bank. Nucleic Acids Res..

[46] Wang T., Cai S., Cheng Y., Zhang W., Wang M., Sun H., Guo B., Li Z., Xiao Y., Jiang S. (2022). Discovery of Small-Molecule Inhibitors of the PD-1/PD-L1 Axis That Promote PD-L1 Internalization and Degradation. J. Med. Chem..

[47] Pence H.E., Williams A. (2010). ChemSpider: An Online Chemical Information Resource. J. Chem. Educ..

[48] Kim S., Chen J., Cheng T., Gindulyte A., He J., He S., Li Q., Shoemaker B.A., Thiessen P.A., Yu B. (2023). PubChem 2023 Update. Nucleic Acids Res..

[49] Zak K.M., Grudnik P., Guzik K., Zieba B.J., Musielak B., Dömling A., Dubin G., Holak T.A. (2016). Structural Basis for Small Molecule Targeting of the Programmed Death Ligand 1 (PD-L1). Oncotarget.

[50] Guzik K., Zak K.M., Grudnik P., Magiera K., Musielak B., Törner R., Skalniak L., Dömling A., Dubin G., Holak T.A. (2017). Small-Molecule Inhibitors of the Programmed Cell Death-1/Programmed Death-Ligand 1 (PD-1/PD-L1) Interaction via Transiently Induced Protein States and Dimerization of PD-L1. J. Med. Chem..

[51] Sanner M.F. (1999). Python: A Programming Language for Software Integration and Development. J. Mol. Graph. Model..

[52] Bento A.P., Hersey A., Félix E., Landrum G., Gaulton A., Atkinson F., Bellis L.J., De Veij M., Leach A.R. (2020). An Open Source Chemical Structure Curation Pipeline Using RDKit. J. Cheminform..

[53] O’Boyle N.M., Banck M., James C.A., Morley C., Vandermeersch T., Hutchison G.R. (2011). Open Babel: An Open Chemical Toolbox. J. Cheminform..

[54] Morris G.M., Huey R., Lindstrom W., Sanner M.F., Belew R.K., Goodsell D.S., Olson A.J. (2009). AutoDock4 and AutoDockTools4: Automated Docking with Selective Receptor Flexibility. J. Comput. Chem..

[55] Trott O., Olson A.J. (2010). AutoDock Vina: Improving the Speed and Accuracy of Docking with a New Scoring Function, Efficient Optimization, and Multithreading. J. Comput. Chem..

[56] Guedes I.A., Pereira Da Silva M.M., Galheigo M., Krempser E., De Magalhães C.S., Correa Barbosa H.J., Dardenne L.E. (2024). DockThor-VS: A Free Platform for Receptor-Ligand Virtual Screening. J. Mol. Biol..

[57] Verdonk M.L., Cole J.C., Hartshorn M.J., Murray C.W., Taylor R.D. (2003). Improved Protein–Ligand Docking Using GOLD. Proteins.

[58] Pawar S.S., Rohane S.H. (2021). Review on Discovery Studio: An Important Tool for Molecular Docking. Asian J. Res. Chem..

[59] Humphrey W., Dalke A., Schulten K. (1996). VMD: Visual Molecular Dynamics. J. Mol. Graph..

[60] Jendele L., Krivak R., Skoda P., Novotny M., Hoksza D. (2019). PrankWeb: A Web Server for Ligand Binding Site Prediction and Visualization. Nucleic Acids Res..

[61] Mysinger M.M., Carchia M., Irwin J.J., Shoichet B.K. (2012). Directory of Useful Decoys, Enhanced (DUD-E): Better Ligands and Decoys for Better Benchmarking. J. Med. Chem..

[62] Case D.A., Aktulga H.M., Belfon K., Cerutti D.S., Cisneros G.A., Cruzeiro V.W.D., Forouzesh N., Giese T.J., Götz A.W., Gohlke H. (2023). AmberTools. J. Chem. Inf. Model..

[63] Wang J., Wang W., Kollman P.A., Case D.A. (2006). Automatic Atom Type and Bond Type Perception in Molecular Mechanical Calculations. J. Mol. Graph. Model..

[64] Izadi S., Anandakrishnan R., Onufriev A.V. (2014). Building Water Models: A Different Approach. J. Phys. Chem. Lett..

[65] Tian C., Kasavajhala K., Belfon K.A.A., Raguette L., Huang H., Migues A.N., Bickel J., Wang Y., Pincay J., Wu Q. (2020). ff19SB: Amino-Acid-Specific Protein Backbone Parameters Trained against Quantum Mechanics Energy Surfaces in Solution. J. Chem. Theory Comput..

[66] Salomon-Ferrer R., Götz A.W., Poole D., Le Grand S., Walker R.C. (2013). Routine Microsecond Molecular Dynamics Simulations with AMBER on GPUs. 2. Explicit Solvent Particle Mesh Ewald. J. Chem. Theory Comput..

[67] Elber R., Ruymgaart A.P., Hess B. (2011). SHAKE Parallelization. Eur. Phys. J. Spec. Top..

[68] Metropolis N., Ulam S. (1949). The Monte Carlo Method. J. Am. Stat. Assoc..

[69] Miller B.R., McGee T.D., Swails J.M., Homeyer N., Gohlke H., Roitberg A.E. (2012). *MMPBSA.Py*: An Efficient Program for End-State Free Energy Calculations. J. Chem. Theory Comput..

[70] Wang E., Sun H., Wang J., Wang Z., Liu H., Zhang J.Z.H., Hou T. (2019). End-Point Binding Free Energy Calculation with MM/PBSA and MM/GBSA: Strategies and Applications in Drug Design. Chem. Rev..

[71] Roe D.R., Cheatham T.E. (2013). PTRAJ and CPPTRAJ: Software for Processing and Analysis of Molecular Dynamics Trajectory Data. J. Chem. Theory Comput..

[72] Kaplan K., Dwivedi P., Davidson S., Yang Q., Tso P., Siems W., Hill H.H. (2009). Monitoring Dynamic Changes in Lymph Metabolome of Fasting and Fed Rats by Electrospray Ionization-Ion Mobility Mass Spectrometry (ESI-IMMS). Anal. Chem..

[73] Daina A., Michielin O., Zoete V. (2017). SwissADME: A Free Web Tool to Evaluate Pharmacokinetics, Drug-Likeness and Medicinal Chemistry Friendliness of Small Molecules. Sci. Rep..

[74] Pires D.E.V., Blundell T.L., Ascher D.B. (2015). pkCSM: Predicting Small-Molecule Pharmacokinetic and Toxicity Properties Using Graph-Based Signatures. J. Med. Chem..

[75] Vattanasit U., Navasumrit P., Khadka M.B., Kanitwithayanun J., Promvijit J., Autrup H., Ruchirawat M. (2014). Oxidative DNA Damage and Inflammatory Responses in Cultured Human Cells and in Humans Exposed to Traffic-Related Particles. Int. J. Hyg. Environ. Health.

[76] Shan K., Liu C., Liu B.-H., Chen X., Dong R., Liu X., Zhang Y.-Y., Liu B., Zhang S.-J., Wang J.-J. (2017). Circular Noncoding RNA HIPK3 Mediates Retinal Vascular Dysfunction in Diabetes Mellitus. Circulation.

[77] Lu K., Li W., Liu X., Sun M., Zhang M., Wu W., Xie W., Hou Y. (2013). Long Non-Coding RNA MEG3 Inhibits NSCLC Cells Proliferation and Induces Apoptosis by Affecting P53 Expression. BMC Cancer.

