# Supplementary figures and images for: A Comparative Molecular Dynamics Study of Food-Derived Compounds as PD-L1 Inhibitors: Insights Across Six Flavonoid Subgroups
Source: Molecules. 2025 Feb 15;30(4):907. doi: 10.3390/molecules30040907 (PMC11858612; doi:10.3390/molecules30040907)

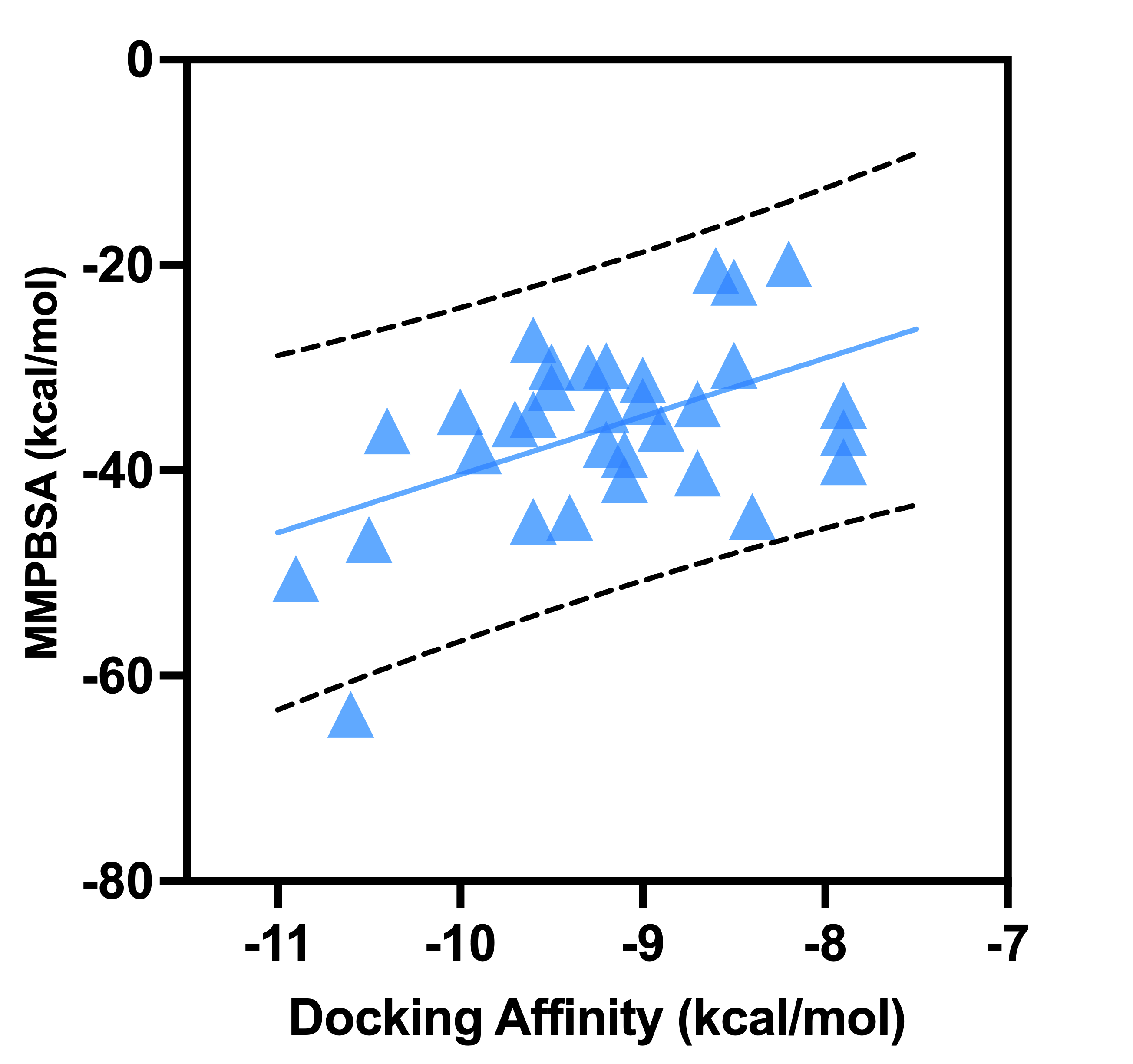

Supplement: Supplementary file 1 [file molecules-30-00907-s001.zip › Figure S1.tiff]

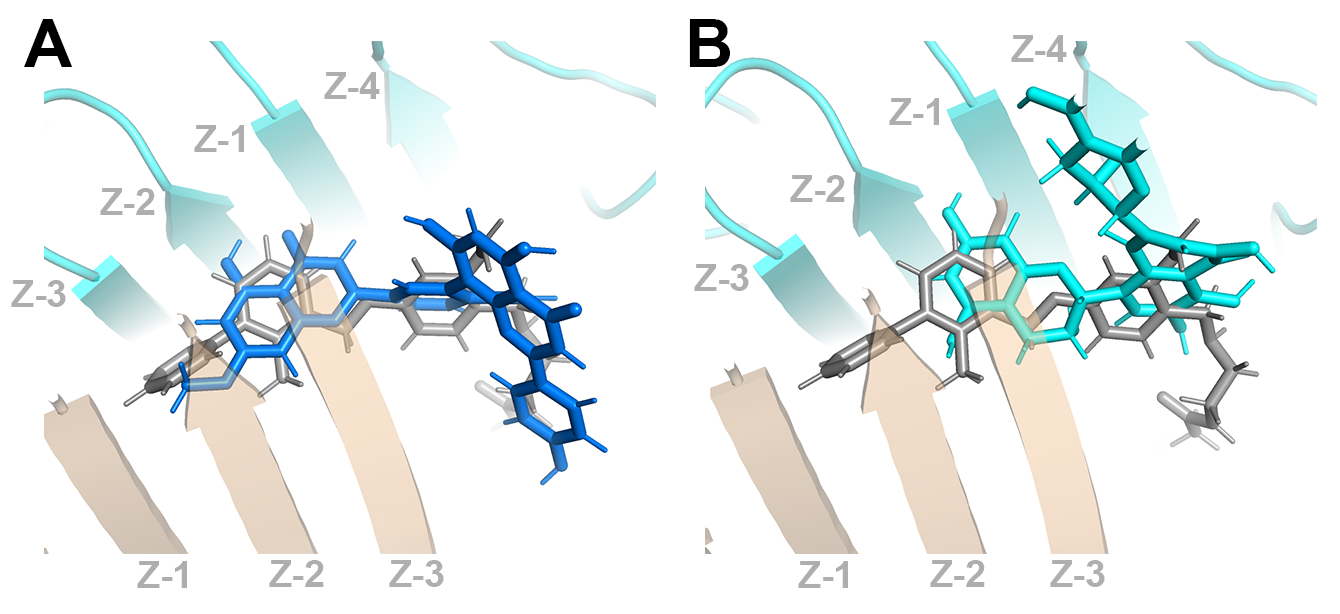

Supplement: Supplementary file 1 [file molecules-30-00907-s001.zip › Figure S2.tiff]

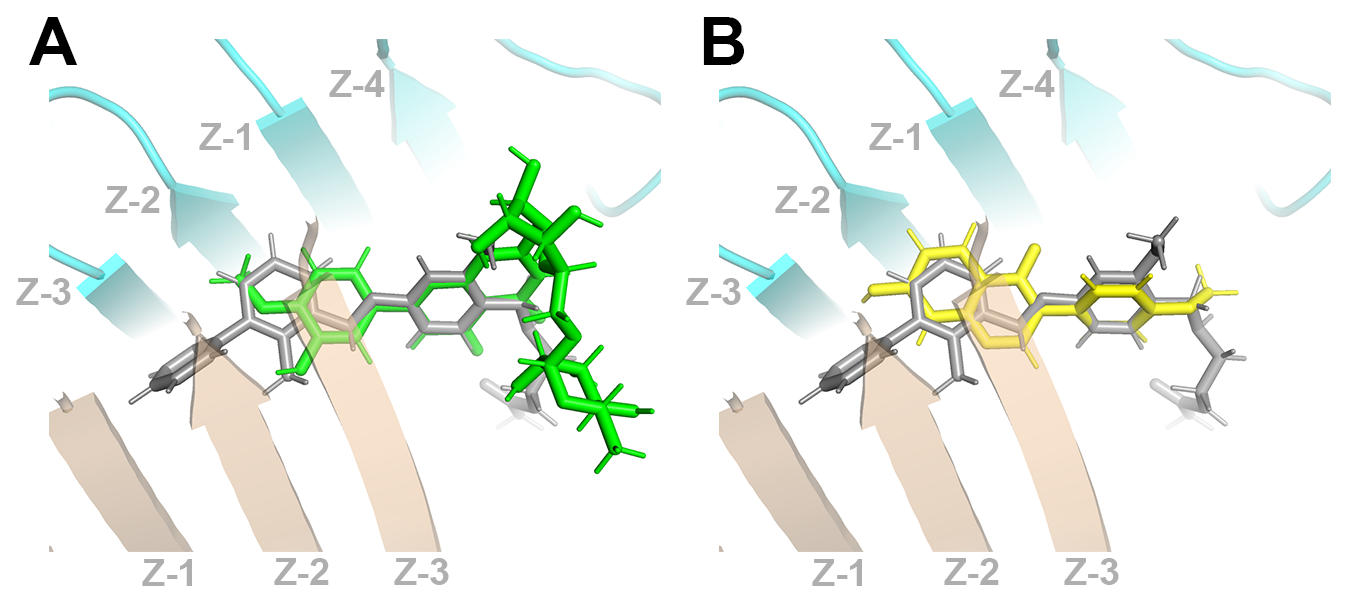

Supplement: Supplementary file 1 [file molecules-30-00907-s001.zip › Figure S3.tiff]

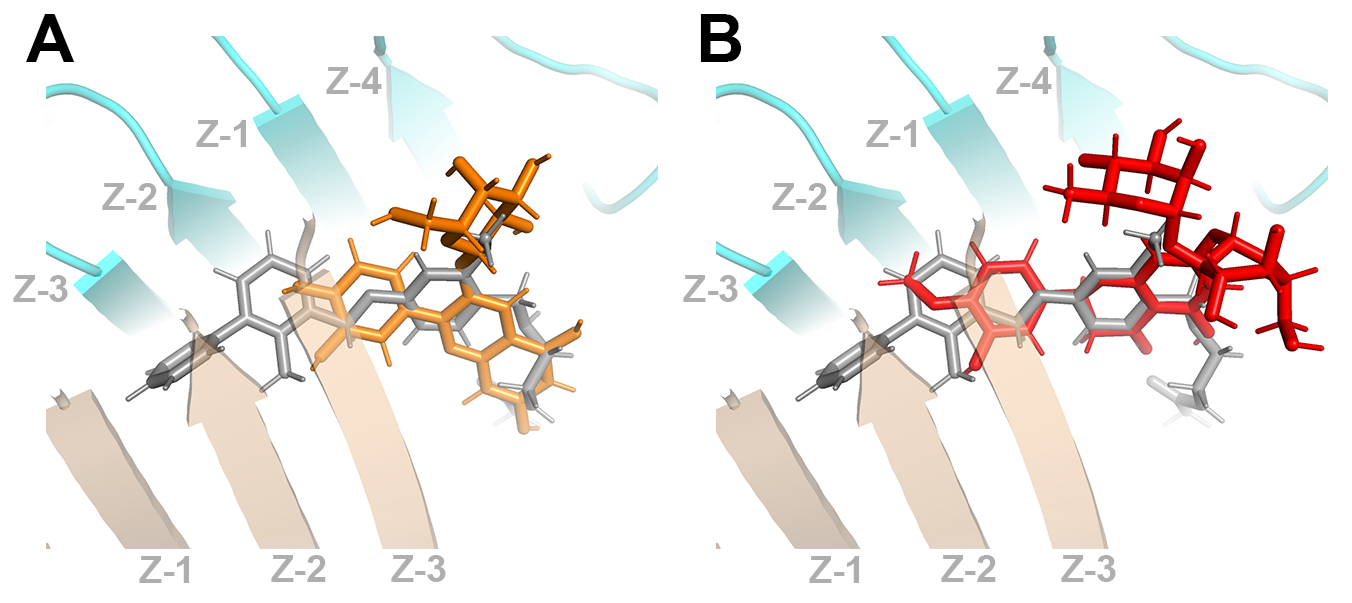

Supplement: Supplementary file 1 [file molecules-30-00907-s001.zip › Figure S4.tiff]

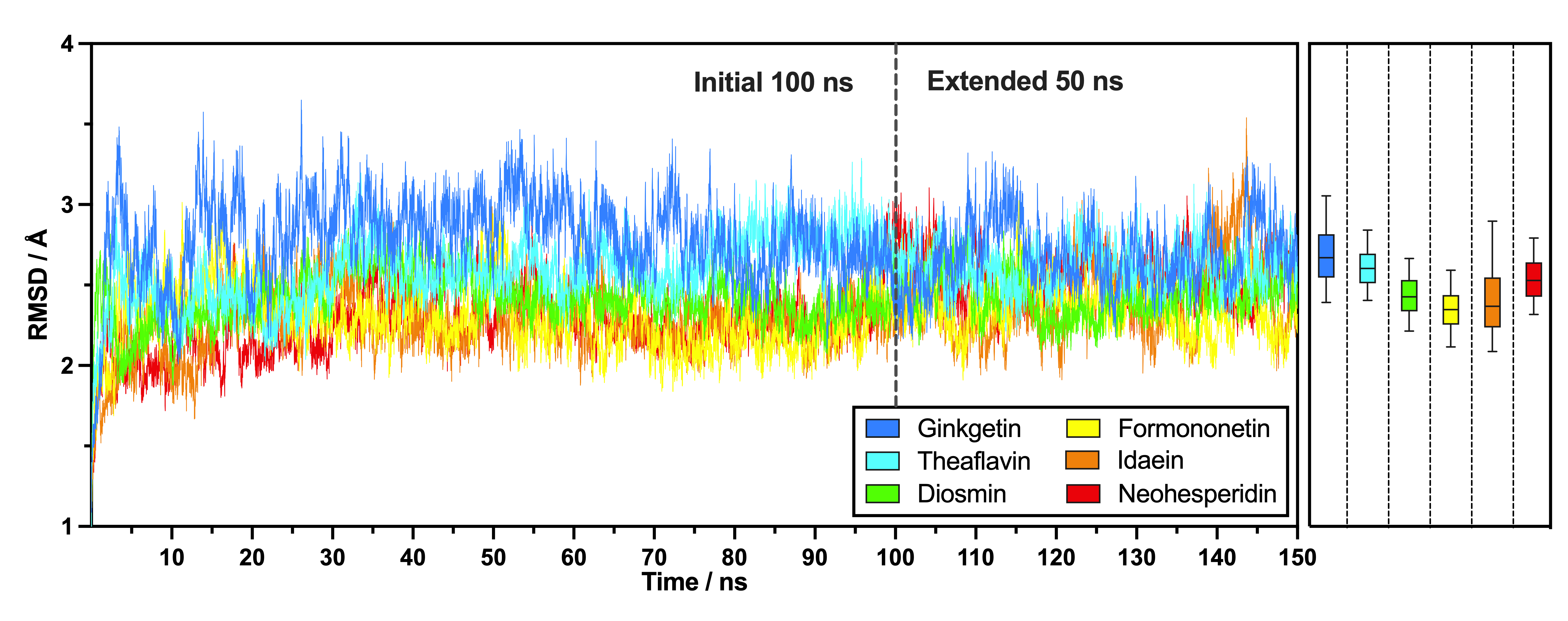

Supplement: Supplementary file 1 [file molecules-30-00907-s001.zip › Figure S5.tiff]

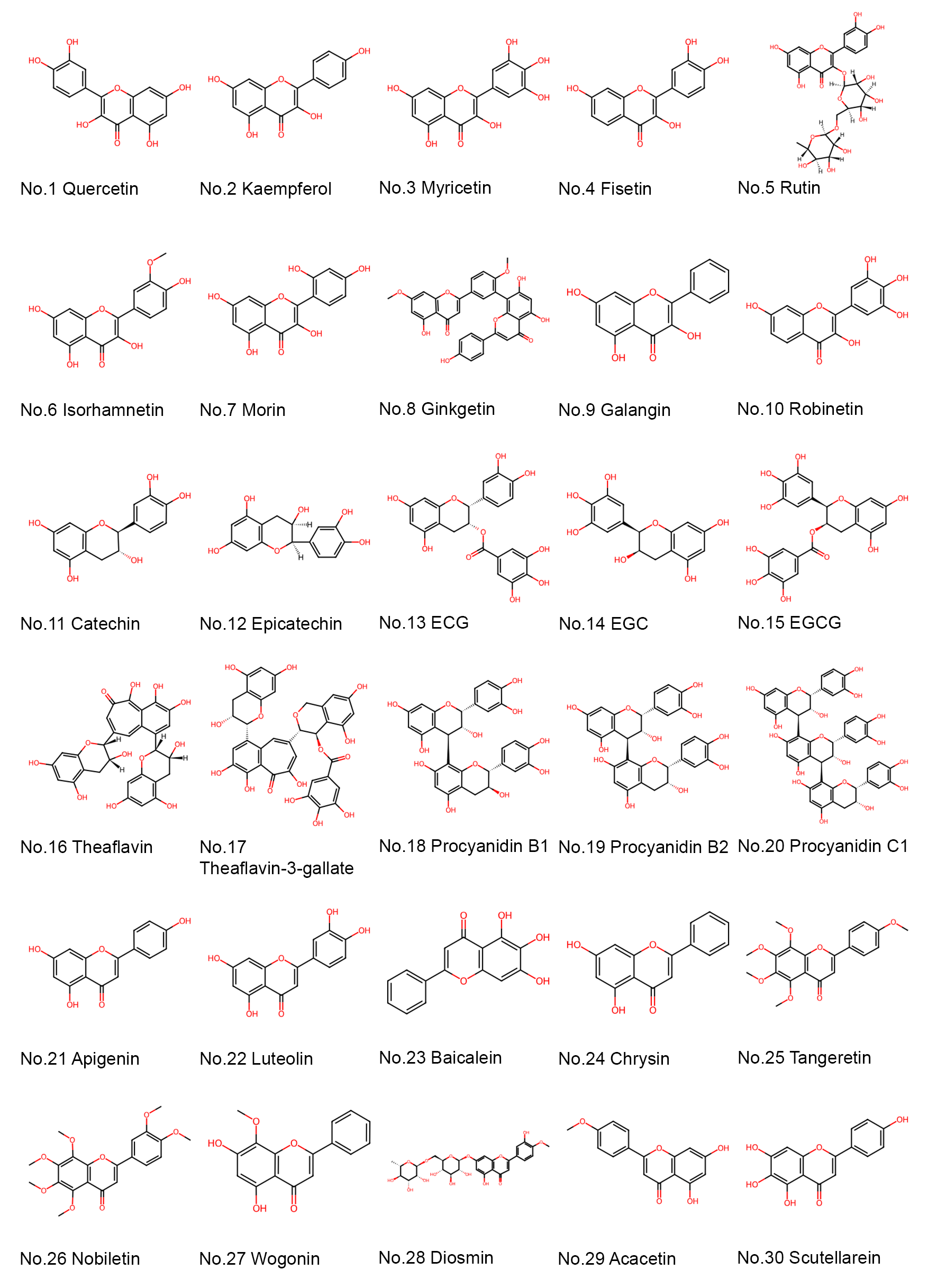

Supplement: Supplementary file 1 [file molecules-30-00907-s001.zip › Figure S6.tiff]

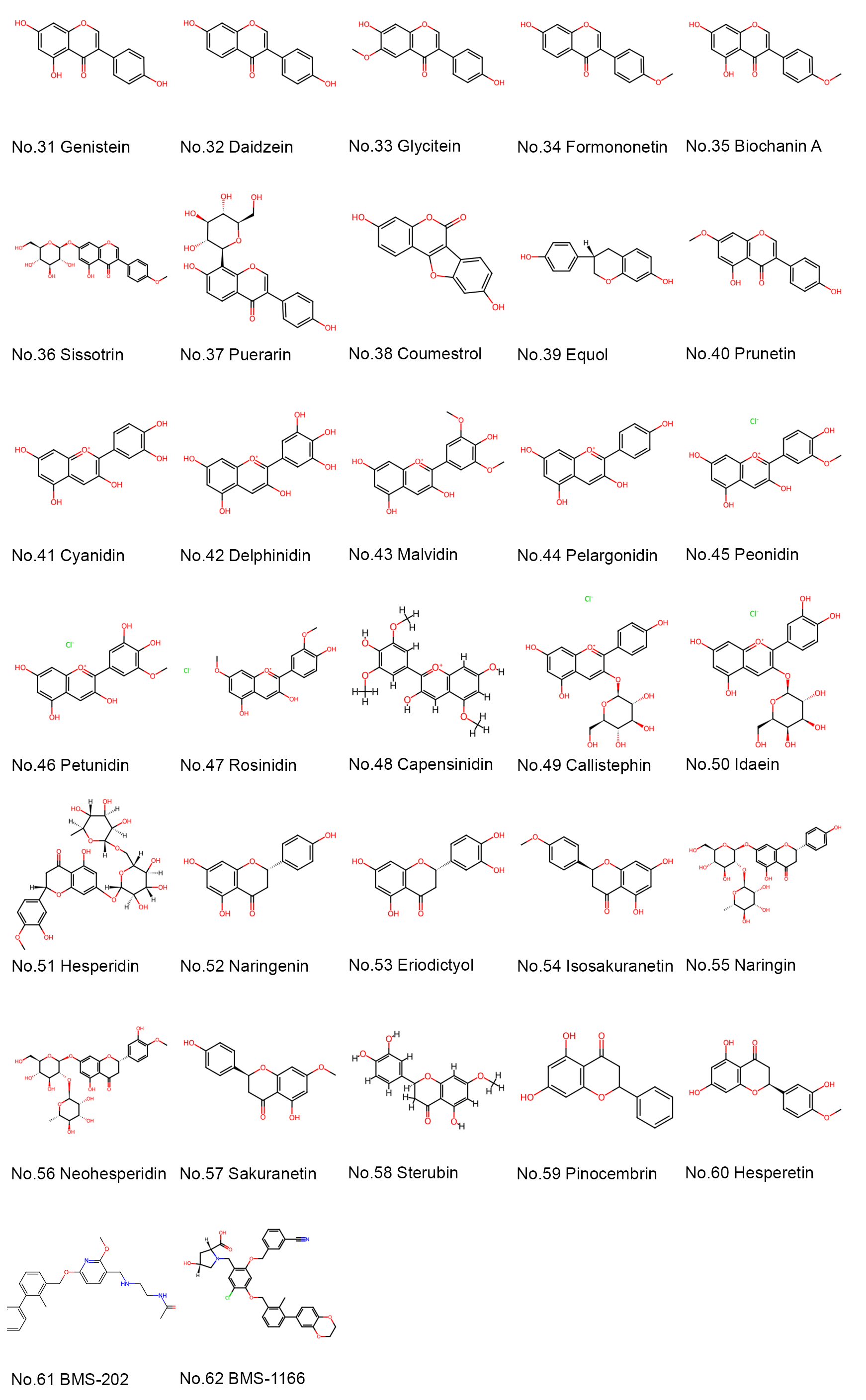

Supplement: Supplementary file 1 [file molecules-30-00907-s001.zip › Figure S7.tiff]

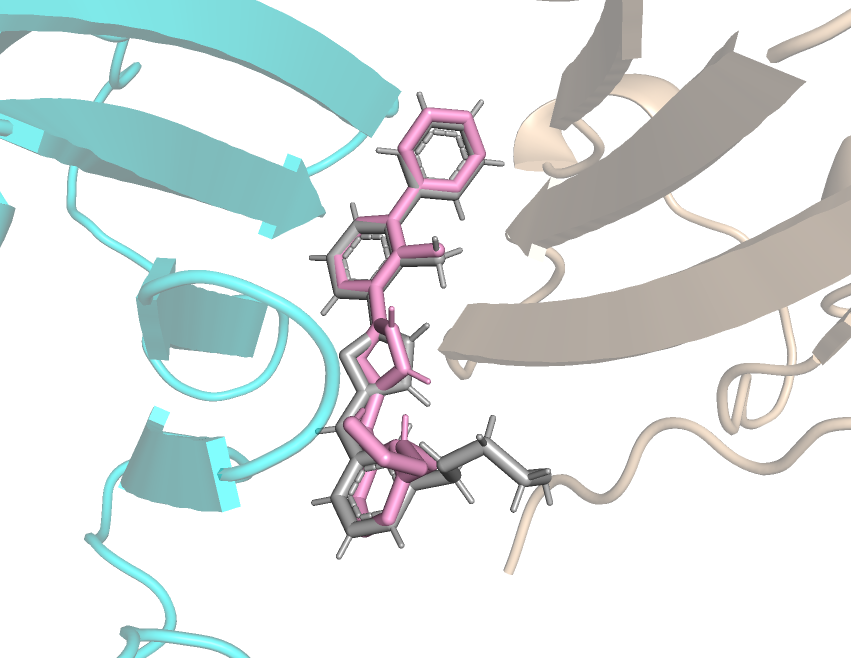

Supplement: Supplementary file 1 [file molecules-30-00907-s001.zip › Figure S8.tiff]

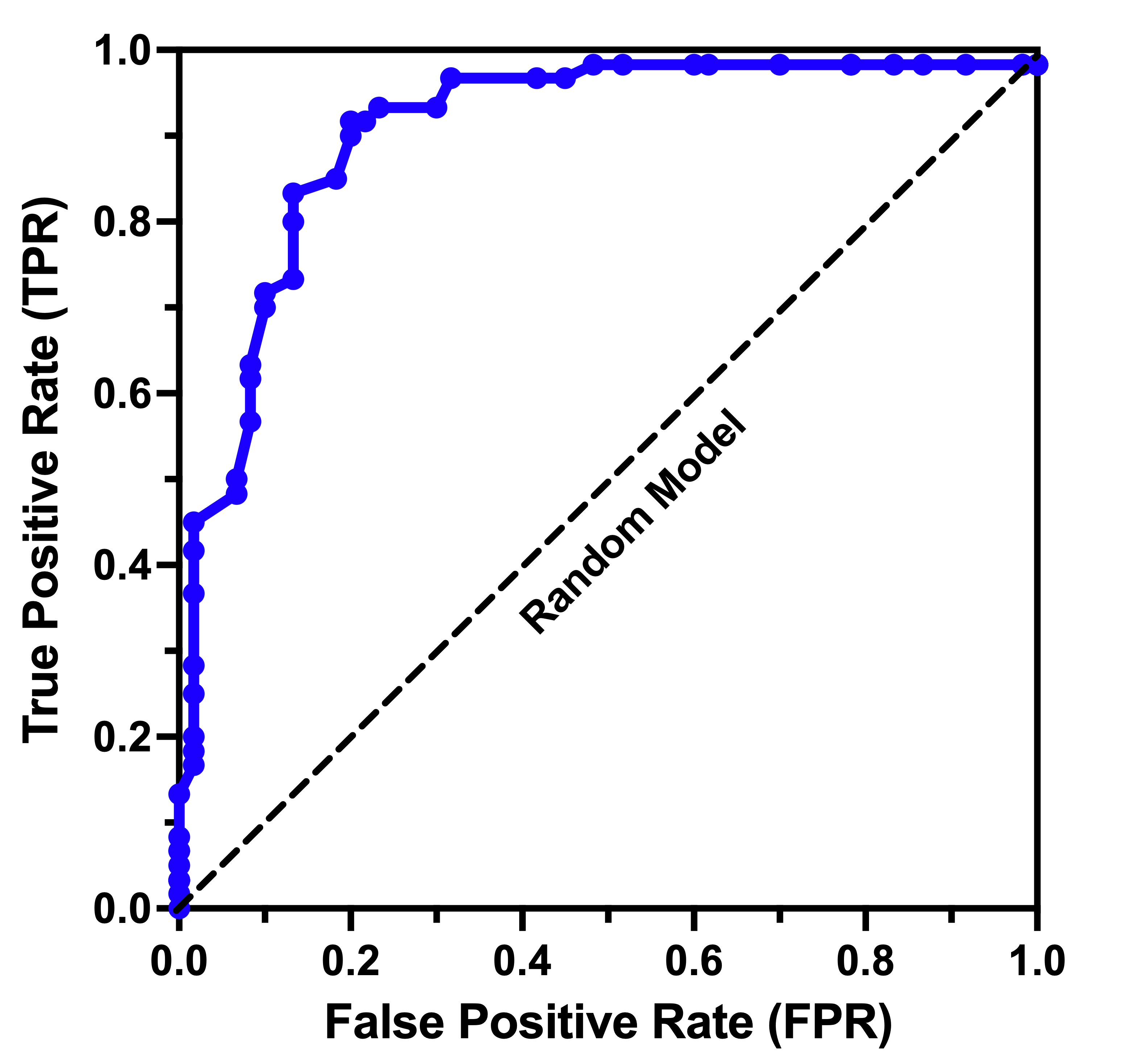

Supplement: Supplementary file 1 [file molecules-30-00907-s001.zip › Figure S9.tiff]

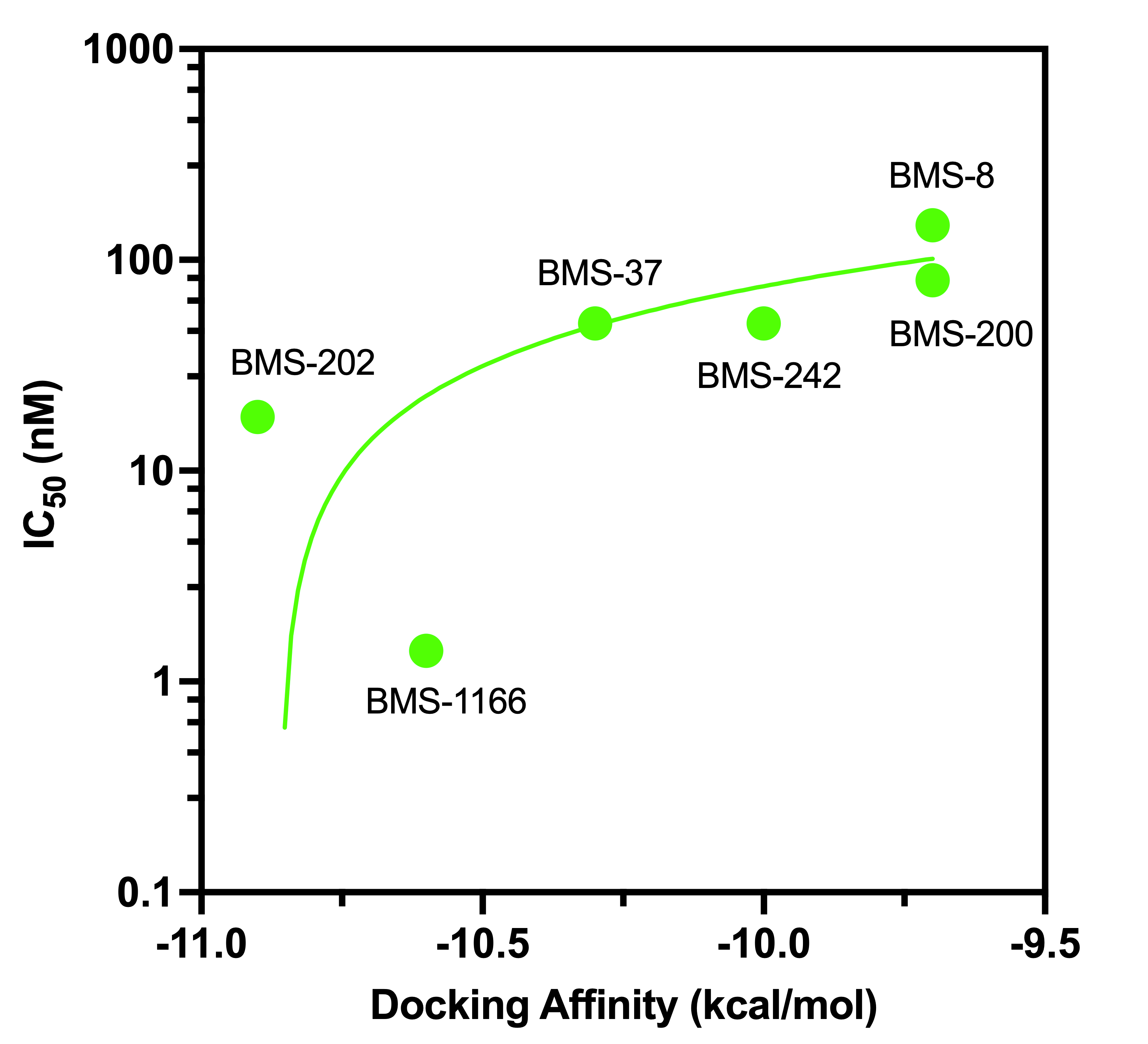

Supplement: Supplementary file 1 [file molecules-30-00907-s001.zip › Figure S10.tiff]
